# Comparative Genomics of *Prunus*-Associated Members of the *Pseudomonas syringae* Species Complex Reveals Traits Supporting Co-evolution and Host Adaptation

**DOI:** 10.3389/fmicb.2022.804681

**Published:** 2022-05-03

**Authors:** Michela Ruinelli, Jochen Blom, Theo H. M. Smits, Joël F. Pothier

**Affiliations:** ^1^Environmental Genomics and Systems Biology Research Group, Institute for Natural Resources Sciences, Zurich University of Applied Sciences (ZHAW), Wädenswil, Switzerland; ^2^Bioinformatics and Systems Biology, Justus-Liebig-University Giessen, Giessen, Germany

**Keywords:** *Pseudomonas syringae* species complex, comparative genomics, pathogenicity, co-evolution, host adaptation

## Abstract

Members of the *Pseudomonas syringae* species complex cause symptoms that are ranging from leaf spots to cankers on a multitude of plant species, including some of the genus *Prunus*. To date, a total of two species of the *P. syringae* species complex and six different pathovars have been associated with diseases on *Prunus* spp., which were shown to belong to different phylogenetic units (phylogroups, PG) based on sequence similarity of housekeeping genes or whole genomes, suggesting that virulence to *Prunus* spp. may be the result of convergent pathoadaptation. In this study, a comparative genomics approach was used to determine genes significantly associated with strains isolated from *Prunus* spp. across a phylogeny of 97 strains belonging to the *P. syringae* species complex. Our study revealed the presence of a set of orthologous proteins which were significantly associated with strains isolated from *Prunus* spp. than in strains isolated from other hosts or from non-agricultural environments. Among them, the type III effector HopAY predicted to encode for a C58 cysteine protease was found to be highly associated with strains isolated from *Prunus* spp. and revealed patterns supporting co-evolution and host adaptation.

## Introduction

Members of the *Pseudomonas syringae* species complex are responsible for the development of plant disease-causing blights, spots, specks, galls, and cankers on a wide range of economically important plant species including both herbaceous and woody hosts. Strains belonging to the *P. syringae* species complex have also been isolated from non-agricultural habitats, and therefore, their persistence and transmission is probably linked to the water cycle (Morris et al., 2008). Despite the economic and ecological importance of this bacterium, the taxonomy and nomenclature of strains belonging to the *P. syringae* species complex is quite confusing and remains largely unsettled (Palleroni, 2005; Gomila et al., 2017). Within the *P. syringae* species complex, at least nine independent species have been determined based on phenotypical and molecular characteristics while more than 60 pathovars have been defined based on the host range (Dye et al., 1980; Palleroni, 2005; Young, 2010). DNA–DNA hybridization experiments among strains belonging to 48 different pathovars of *P. syringae* revealed the existence of nine different genomospecies (Gardan et al., 1999), which were later reflected by the so-called phylogroups (PG) obtained based on sequence similarity of housekeeping genes (Sarkar and Guttman, 2004; Hwang et al., 2005; Sarkar et al., 2006; Parkinson et al., 2011). With the inclusion of strains isolated from non-agricultural environments, a total of 13 PG were defined (Berge et al., 2014).

Many studies have been performed in the last decades with the intent to investigate and determine factors related to pathogenicity of *P. syringae* strains. The presence of the *hypersensitive reaction* and *pathogenicity* (*hrp*)/*hypersensitive reaction* and *conserved* (*hrc*) cluster was shown to be essential for pathogenicity of *P. syringae* pv. phaseolicola on bean and for triggering hypersensitive response (HR) on non-host plants, such as tobacco and tomato (Lindgren et al., 1986, 1988; Bogdanove et al., 1996). A homologous region with similar function was found also in other plant pathogens (Beer et al., 1991; Bonas et al., 1991; Bogdanove et al., 1996) and was later shown to encode for a type III secretion system (T3SS) with homology to the virulence protein secretion system (Yop) of animal-pathogenic *Yersinia* spp. (Gough et al., 1992). In *P. syringae*, the T3SS encodes for a protein apparatus which is responsible for the delivery of virulence-related factors, so-called type III effectors (T3E), into the plant cell (Wei et al., 2000). T3E generally act by promoting pathogenicity or by suppressing host immune defense but constitute a double-edge sword since T3E can also be recognized by specific plant resistance proteins which in turn trigger host immune system (Mackey et al., 2002; Shao et al., 2003; Xiang et al., 2008). However, many T3E have been shown to be functionally redundant thus decreasing the selective pressure on the host to evolve resistance proteins against single T3E (Kvitko et al., 2009). This observation suggested that the compatible interaction between *P. syringae* and its host is defined by the totality of its T3E repertoire (Lindeberg et al., 2012).

With the advent of affordable next-generation sequencing technologies, many complete and draft genome sequences of strains belonging to the *P. syringae* species complex have become available. Comparative genomics studies within different pathovars of the *P. syringae* species complex also revealed that adaptation to woody hosts was reflected by the presence of genes involved in the degradation of woody plant species-related compounds like the pentose sugar xylose and aromatic compounds, such as toluene and catechol (Green et al., 2010; Bartoli et al., 2015; Caballo-Ponce et al., 2016; Nowell et al., 2016; Hulin et al., 2020). Many studies have focused on the determination of the T3E repertoire of strains isolated from different hosts (Lindeberg et al., 2012; Ruinelli et al., 2019) and it is only recently that a few of them reported the convergent acquisition of T3E in strains adapted to the same host (Hulin et al., 2018; Newberry et al., 2019; Moreno-Pérez et al., 2020) or that strain differences in T3E alleles could be linked to host specificity (Zembek et al., 2018; Jayaraman et al., 2020). These findings underline the importance of whole genome-based comparisons to investigate factors involved in the host–pathogen interactions, which indeed are more complex than initially thought.

The plant genus *Prunus* includes economically important stone fruit trees, such as sweet cherry (*Prunus avium*), sour cherry (*Prunus cerasus*), and peach (*Prunus persica*), which in 2018 accounted for 11.6% of the total fruit orchard area in Europe (Eurostat, 2018). Even more important for the European market are almond trees (*Prunus amygdalus*) which in 2018 occupied as single species 22.6% of the total area dedicated to growing fruits (Eurostat, 2018). Bacterial canker on *Prunus* spp. caused by members of the *P. syringae* species complex affects all aboveground organs of the tree causing heavy yield reduction (up to 75%) and can lead to death of the whole tree, especially in young orchards (Crosse, 1966; Spotts et al., 2010; Hulin et al., 2020). Typical symptoms visible on trunks and branches include sunken, dark brown dieback, and cankers, which are sometimes accompanied by gummy leaks (Puławska et al., 2017). Blossom wilting and browning is mainly visible on highly susceptible varieties and constitute an important source of secondary infection. In addition, necrotic spots can be observed on leaves and on fruits which then lose their commercial values (Puławska et al., 2017). Within the *P. syringae* species complex, three different PG contain two *Pseudomonas* species and six *P. syringae* pathovars, which were found in association with diseases on *Prunus* spp.

Bacterial canker of sweet and sour cherry is mainly caused by strains belonging to *P. syringae* pv. morsprunorum race 1 and *P. syringae* pv. morsprunorum race 2 (Crosse, 1959; Crosse and Garrett, 1963; Freigoun and Crosse, 1975; Ruinelli et al., 2019). Despite being classified as races of the same pathovar, phylogenetic analysis based on sequence similarity of four housekeeping genes or of core genome of 2,085 coding sequences revealed that strains of the *P. syringae* pv. morsprunorum race 1 belong to PG3, whereas strains of the *P. syringae* pv. morsprunorum race 2 cluster within PG1 (Nowell et al., 2016; Ruinelli et al., 2019), underlying the need for clarification of the nomenclature of members of the *P. syringae* species complex.

Bacterial dieback of peach is caused by *P. syringae* pv. persicae (PG1; Young, 1987) which is also causes disease on nectarine and is weakly pathogenic to plum but not causing disease on apricot and cherry (Young, 1987). Due to its limited distribution in Europe, *P. syringae* pv. persicae was classified as quarantine organism from the European and Mediterranean Plant Protection Organization (EPPO, 2005) and as recommended regulated non-quarantine pest in the EU plant health regulation in force since December 2019 (Picard et al., 2018). Strains belonging to the *P. syringae* pv. avii (PG1) were isolated from wild cherry trees (*Prunus avium*) affected by bacterial canker in France and were shown to be only weakly pathogenic to peach, plum, and apricot (Ménard et al., 2003). *Pseudomonas amygdali* and *P. syringae* pv. cerasicola, both belonging to PG3, are the causal agents of the bacterial hyperplastic canker of almond (*P. amygdalus*; Psallidas, 1997) and bacterial gall of ornamental cherry (*Prunus* × *yedoensis*; Kamiunten et al., 2000), respectively. A few years ago, a new species belonging to PG2, namely, *Pseudomonas cerasi* (Kałużna et al., 2016), was found to be responsible for the development of bacterial canker on cherry trees in Poland and more recently on pear tree in South Korea (Choi et al., 2020).

In addition, symptoms of bacterial canker on *Prunus* spp. are also caused by strains of *P. syringae* pv. syringae belonging to PG2 (Crosse and Garrett, 1966). However, in contrast to all above-mentioned pathovars which have been specifically found in association with plant species belonging to the genus *Prunus*, strains of *P. syringae* pv. syringae display a broader host range and are responsible for diseases on many other woody and herbaceous hosts (Cazorla et al., 1998; Garibaldi et al., 2007; Zhou et al., 2012; Popović et al., 2015; Ivanović et al., 2017).

In this study, a comparative genomics approach was used to investigate factors potentially involved in the adaptation of *P. syringae* to plant species belonging to the *Prunus* genus. Our study revealed the presence of a set of orthologous proteins, which were significantly more present in strains isolated from *Prunus* spp. than in strains isolated from other hosts or environments. Among them, the T3E HopAY, potentially encoding for a C58 cysteine protease was found to be highly associated with strains isolated from *Prunus* spp. and revealed patterns supporting co-evolution and host adaptation.

## Materials and Methods

### Phylogenomics

For comparative genomics purpose, the whole genomes data of 97 strains belonging to the *P. syringae* species complex, together with one *P. fluorescens* (strain Pf0-1) and one *P. putida* (strain KT2440) were used (Table 1). A total of 20 genomes were complete and 79 were draft. The selected set of *P. syringae* genomes consisted of strains isolated from plants (*n* = 81) as well as strains isolated from non-agricultural environments (*n* = 15) and represents 11 of the 13 PG defined by Berge et al. (2014). Plant-associated strains were isolated from over 30 different plant species comprising *Prunus* spp. (*n* = 20), *Actinidia chinensis* (*n* = 4), *Solanum lycopersicum* (*n* = 4), *Corylus avellana* (*n* = 5), *Cucumis* spp. (*n* = 5), *Aesculus hippocastanum* (*n* = 3), *Triticum aestivum* (*n* = 3), *Hordeum vulgare* (*n* = 3), *Phaseolus vulgaris* (*n* = 2), *Olea europaea* (*n* = 2), *Glycine max* (*n* = 2), *Nicotiana* sp. (*n* = 2), *Pyrus* sp. (*n* = 2), and other herbaceous and woody hosts (*n* = 22). Non-annotated genomes retrieved from the NCBI database were annotated using a command line annotation pipeline based on HMMer against an EDGAR based database of *Pseudomonas* ortholog groups followed by reference genome annotation and a comparison to the Swiss-Prot and RefSeq databases for genes that had no high-quality hit in previous steps (Linke et al., 2011).

**Table 1:** List of strains used for this study.

Strain^1^	Code	Origin^2^	Host	GenBank accession^3^	Reference^4^	Genome subset
*P. syringae* pv. avii CFBP 3846^P^	Pavii CFBP 3846	FR, 1991	*Prunus avium*	LT963402- LT963407	Ruinelli et al., 2019	A
*P. syringae* pv. persicae CFBP 1573^P^	Ppe CFBP 1573	FR, 1974	*Prunus persica*	ODAL01	Ruinelli et al., 2019	A
*P. syringae* pv. persicae NCPPB 2254	Ppe NCPPB 2254	FR, 1969	*P. persica*	ODAM01	Ruinelli et al., 2019	A
*P. syringae* pv. persicae NCPPB 2254^*^	Ppe NCPPB 2254^*^	FR, 1969	*P. persica*	LAZV01	Zhao et al., 2015	–
*P*. *amygdali* pv. morsprunorum race 2 HRI W 5261	Pmp2 HRIW5261	UK, 1990	*P. avium*	LIIA01	Nowell et al., 2016	B
*P*. *syringae* pv. morsprunorum race 2 CFBP 3800	Pmp2 CFBP 3800	UK, N.D.	*Prunus cerasus*	OLMQ01	Ruinelli et al., 2019	B
*P*. *syringae* pv. morsprunorum race 2 CFBP 6411	Pmp2 CFBP 6411	UK, 1995	*P. avium*	LT963408	Ruinelli et al., 2019	B
*P*. *amygdali* pv. morsprunorum race 2 MAFF 302280^P^	Pmp2 MAFF 302280	US, N.D.	*Prunus domestica*	AEAE01	Baltrus et al., 2011	B
*P*. *cerasi* PL58^T^	P. cerasi PL58	PL, 2007	*P. cerasus*	LT222313- LT222319	Kałużna et al., 2016	D
*P*. *cerasi* PL963	P. cerasi PL963	PL, 2007	*P. avium*	LT963395- LT963400	Ruinelli et al., 2019	D
*P*. *syringae* pv. syringae 2339	Psy 2339	HU, 1984	*P. avium*	LIHU01	Nowell et al., 2016	–
*P*. *syringae* pv. syringae CFBP 2118	Psy CFBP 2118	FR, 1979	*P. cerasus*	LT962481	Ruinelli et al., 2019	–
*P*. *syringae* pv. syringae CFBP 4215	Psy CFBP 4215	FR. 1997	*P. avium*	LT962480	Ruinelli et al., 2019	–
*P*. *amygdali* CFBP 3205^T^	P. amygdali CFBP 3205	GR, 1967	*Prunus amygdalus*	JYHB01	Bartoli et al., 2015	–
*P*. *amygdali* pv. morsprunorum race 1 2341	Pmp1 2341	HU, 1988	*P. cerasus*	LIIB01	Nowell et al., 2016	C
*P*. *amygdali* pv. morsprunorum FTRS U7805^*^	Pmp FTRSU7805^*^	JP, 1978	*Prunus mume*	LGLQ01	N.A.	–
*P*. *amygdali* pv. morsprunorum race 1 HRI W 5269	Pmp1 HRIW5269	UK, 1990	*P. cerasus*	LIHZ01	Nowell et al., 2016	C
*P*. *syringa*e pv. morsprunorum race 1 CFBP 2116	Pmp1 CFBP 2116	FR, 1974	*P. cerasus*	LT985192- LT985195; OLMD01	Ruinelli et al., 2019	C
*P*. *syringae* pv. morsprunorum race 1 CFBP 3840	Pmp1 CFBP 3840	FR, 1996	*P. avium*	LT963409- LT963413	Ruinelli et al., 2019	C
*P*. *syringae* pv. cerasicola CFBP 6109^P^	Pscer CFBP 6109	JP, 1995	*Prunus × yedoensis*	LT963391- LT963394	Ruinelli et al., 2019	C
*P*. *syringae* pv. cerasicola CFBP 6110	Pscer CFBP 6110	JP, 1995	*Prunus × yedoensis*	LT985210- LT985212; OLMP01	Ruinelli et al., 2019	C
*P*. *syringae* pv. actinidiae biovar 3 ICMP 18884	Psa3 ICMP 18884	NZ, 2010	*Actinidia chinensis*	CP011972- CP011973	Templeton et al., 2015	B
*P*. *syringae* pv. actinidiae biovar 2 ICMP 19073	Psa2 ICMP 19073	KR, 1998	*A. chinensis*	AOJR01	McCann et al., 2013	B
*P*. *syringae* pv. actinidiae biovar 1 ICMP 9617^P^	Psa1 ICMP 9617	JP, 1984	*A. chinensis*	CM002753- CM002754	McCann et al., 2013	B
*P*. *syringae* pv. actinidifoliorum ICMP 18883	Pfm ICMP 18883	NZ, 2010	*A. chinensis*	AOKH01	McCann et al., 2013	B
*P*. *syringae* pv. tomato DC3000	Pto DC3000	UK, 1960	*Solanum lycopersicum*	AE016853- AE016855	Buell et al., 2003	A
*P*. *syringae* pv. tomato NCPPB 1108	Pto NCPPB 1108	UK, 1960	*S. lycopersicum*	ADGA01	N.A.	A
*P*. *syringae* pv. tomato NYS-T1	Pto NYS-T1	US, 2009	*S. lycopersicum*	JRRA01	Jones et al., 2015	A
*P*. *syringae* pv. tomato T1	Pto T1	1986	*S. lycopersicum*	ABSM01	Almeida et al., 2009	A
*P*. *avellanae* BPIC 631^T^	Pav BPIC631	GR, 1976	*Corylus avellana*	AKBS01	O'Brien et al., 2012	B
*P*. *avellanae* CRAFRUEC1	Pav CRAFRUEC1	IT, 2003	*C. avellana*	ATLL01	Scortichini et al., 2013	B
*P*. *avellanae* PaVt10	Pav PaVt10	IT, 2010	*C. avellana*	JYHC01	Bartoli et al., 2015	B
*P*. *syringae* pv. avellanae ISPAVE013	Psav ISPAVE013	IT, 1992	*C. avellana*	AKCJ01	O’Brien et al., 2012	–
*P*. *syringae* pv. avellanae ISPAVE037	Psav ISPAVE037	IT, 1992	*C. avellana*	AKCK01	O'Brien et al., 2012	–
*P*. *amygdali* pv. lachrymans MAFF 302278	Pla M302278	US, 1935	*Cucumis sativus*	AEAM01	Baltrus et al., 2011	A
*P*. *syringae* CC440	CC440	FR, 2002	*Cucumis melo*	AVEC02	Baltrus et al., 2014	–
*P*. *syringae* CC457	CC457	FR, 2003	*C. melo*	AVEB02	Baltrus et al., 2014	–
*P*. *syringae* CC94	CC94	FR, 1997	*C. melo*	AVEA02	Baltrus et al., 2014	–
*P*. *amygdali* pv. lachrymans MAFF 301315	Pla MAFF 301315	JP, 1975	*C. sativus*	AEAF01	Baltrus et al., 2011	C
*P*. *amygdali* pv. aesculi 2250	Pae 2250	United Kingdom	*Aesculus hippocastanus*	ACXT01	Green et al., 2010	–
*P*. *amygdali* pv. aesculi 0893_23	Pae 0893_23	IN, 1969	*A. hippocastanus*	AEAD01	Baltrus et al., 2011	–
*P*. *amygdali* pv. aesculi NCPPB 3681^P^	Pae NCPPB 3681	IN, 1980	*A. hippocastanus*	ACXS01	Green et al., 2010	–
*P*. *syringae* pv. atrofaciens DSM 50255	Paf DSM 50255	CA, 1942	*Triticum aestivum*	AWUI01	N.A.	–
*P*. *syringae* pv. syringae B64	Psy B64	N.D.	*T. aestivum*	ANZF01	Dudnik and Dudler, 2013b	–
*P*. *syringae* pv. syringae SM	Psy SM	United States	*T. aestivum*	APWT01	Dudnik and Dudler, 2013a	–
*P*. *syringae* BRIP39023	BRIP39023	AU, 1971	*Hordeum vulgare*	AMZX01	Gardiner et al., 2013	D
*P*. *syringae* BRIP34876	BRIP34876	AU, 1971	*H. vulgare*	AMXK01	Gardiner et al., 2013	–
*P*. *syringae* BRIP34881	BRIP34881	AU, 1971	*H. vulgare*	AMXL01	Gardiner et al., 2013	–
*P*. *syringae* pv. syringae B728a	Psy B728a	US, 1987	*Phaseolus vulgaris*	CP000075	Feil et al., 2005	–
*P*. *syringae* pv. phaseolicola 1448a	Pph 1448a	ET, 1985	*P. vulgaris*	CP000058- CP000060	Joardar et al., 2005	C
*P*. *savastanoi* pv. savastanoi DAPP-PG722	Psv DAPP-PG722	IT, 2007	*Olea europaea*	JOJV01	Moretti et al., 2014	–
*P*. *savastanoi* pv. savastanoi PseNe107	Psv PseNe107	NP, 2007	*O. europaea*	JYHF01	Bartoli et al., 2015	–
*P*. *syringae* CC1458	CC1458	US, 2005	*Dodecantheon pulchellum*	AVEN02	Baltrus et al., 2014	–
*P*. *syringae* CC1466	CC1466	US, 2005	*D. pulchellum*	AVEM02	Baltrus et al., 2014	–
*P. savastanoi* pv. glycinea B076	Pgy B076	2007	*Glycine max*	AEGG01	Qi et al., 2011	C
*P. savastanoi* pv. glycinea str. race 4	Pgy r4	1977	*G. max*	AEGH01	Qi et al., 2011	C
*P. amygdali* pv. tabaci 6605	Pta 6605	JP	*Nicotiana* sp.	AJXI01	N.A.	C
*P. amygdali* pv. tabaci ATCC 11528	Pta ATCC 11528	US, 1905	*Nicotiana* sp.	AEAP01	Baltrus et al., 2011	C
*P. syringae* pv. syringae A2	Psy A2	N.D.	*Pyrus calleryana*	LGKU01	N.A.	–
*P. syringae* pv. syringae B301D-R	Psy B301D-R	UK, 1969	*Pyrus communis* L.	JALJ01	Dudnik and Dudler, 2014	–
*P. syringae* CC1630	CC1630	US, 2007	*Onobrychis* sp.	AVED02	Baltrus et al., 2014	–
*P. syringae* pv. maculicola CFBP 1657^P^	Pma CFBP1657	NZ, 1965	*Brassica oleracea*	JYHH01	Bartoli et al., 2015	A
*P. syringae* pv. theae ICMP 3923	Pth ICMP 3923	JP, 1970	*Camellia sinensis*	LJRU01	N.A.	–
*P. syringae* pv. viburni ICMP 3963^P^	Pvi ICMP 3963	US, N.d.	*Viburnum* sp.	LJRR01	N.A.	–
*P. syringae* pv. papulans ICMP 4048^P^	Ppp ICMP 4048	CN, 1973	*Malus × domestica*	LJRB01	N.A.	D
*P. syringae* UMAF0158	UMAF0158	ES, 1993	*Mangifera* sp.	CP005970- CP005971	Martínez-García et al., 2015	D
*P. syringae* pv. panici LMG 2367^P^	Ppa LMG 2367	US, 1963	*Panicum* sp.	ALAC01	Liu et al., 2012	–
*P. syringae* pv. syringae DSM 10604^T^	Psy DSM 10604	UK, 1950	*Syringa vulgaris*	JALK01	N.A.	–
*P. syringae* pv. syringae HS191	Psy HS191	AU, 1979	*Panicum miliaceum*	CP006256- CP006257	Ravindran et al., 2015	–
*P. syringae* pv. syringae 642	Psy 642	US, 2007	Unidentified weed	ADGB01	Clarke et al., 2010	–
*P. syringae* pv. syringae 1212	Psy 1212	United Kingdom	*Pisum sativum*	AVCR02	Baltrus et al., 2014	–
*P. amygdali* pv. dendropanacis CFBP 3226^P^	Pde CFBP 3226	JP, 1979	*Dendropanax trifidus*	JYHG01	Bartoli et al., 2015	–
*P. meliae* CFBP 3225^T^	P.meliae CFBP 3225	JP, 1974	*Melia azedarach*	JYHE01	Bartoli et al., 2015	–
*P. coronafaciens* pv. atropurpurea ICMP 4457^P^	Par ICMP 4457	JP, 1967	*Lolium multiflorum*	LJPS01	N.A.	–
*P. syringae* CC1513	CC1513	FR, 2006	*Hutchinsia alpina*	AVEL02	Baltrus et al., 2014	–
*P. syringae* CC1629	CC1629	US, 2007	*Avena sativa*	AVEE02	Baltrus et al., 2014	–
*P. cannabina* ICMP 2823	Pcb ICMP 2823	HU, 1957	*Cannabis sativa*	LJPX01	N.A.	–
*P. cannabina* pv. alisalensis ES4326	Pal ES4326	US, 1965	*Raphanus sativus*	AEAK01	Baltrus et al., 2011	–
*P. syringae* pv. helianthi ICMP 4531^P^	Phe ICMP4531	MX, 1972	*Helianthus annuus*	LJQM01	N.A.	–
*P. syringae* pv. tagetis ICMP 4091^P^	Ptg ICMP4091	ZW, 1972	*Tagetes erecta*	LJRM01	N.A.	–
*P. viridiflava* TA043	Pvir TA043	FR, 2007	*Primula officinalis*	AVDV01	Baltrus et al., 2014	–
*P. viridiflava* UASWS0038	Pvir UASWS0038	CH, 2007	*Rhododendron* sp.	AMQP01	Lefort et al., 2013	–
*P. syringae* CC1416	CC1416	US, 2004	Epilithon	AVEP02	Baltrus et al., 2014	–
*P. syringae* CC1544	CC1544	FR, 2006	Lake water	AVEI02	Baltrus et al., 2014	–
*P. syringae* CC1559	CC1559	FR, 2006	Snow	AVEG02	Baltrus et al., 2014	–
*P. syringae* USA007	USA007	US, 2007	Stream water	AVDY02	Baltrus et al., 2014	–
*P. syringae* CC1543	CC1543	FR, 2006	Lake water	AVEJ02	Baltrus et al., 2014	–
*P. syringae* UB0390	UB0390	FR,2007	River water	JPQV01	N.A.	–
*P. syringae* UB303	UB303	FR, 2006	Lake water	AVDZ02	Baltrus et al., 2014	–
*P. syringae* USA011	USA011	US, 2007	Stream water	AVDX02	Baltrus et al., 2014	–
*P. viridiflava* CC1582	Pvir CC1582	FR, 2006	Epilithon	AVDW01	Baltrus et al., 2014	–
*P. syringae* CC1417	CC1417	US, 2004	Epilithon	AVEO02	Baltrus et al., 2014	–
*P. syringae* CC1524	CC1524	FR, 2006	Stream water	AVEK02	Baltrus et al., 2014	–
*P. syringae* CC1583	CC1583	FR, 2006	Epilithon	AVEF02	Baltrus et al., 2014	–
*P. syringae* CC1557	CC1557	FR, 2006	Snow	CP007014- CP007015	N.A.	–
*P. syringae* GAW0119	GAW0119	FR, 2010	Irrigation canal	JPQU01	N.A.	–
*P. syringae* CEB003	CEB003	FR, 2010	Stream water	JPQT01	N.A.	–
*P. fluorescens* Pf0-1	Pfl Pf0-1	US, 1987	Soil	CP000094	Silby et al., 2009	–
*P*. *putida* KT2440	Ppu KT2440	N.D.	Soil	AE015451	Nelson et al., 2002	–

1 *Superscript following strain names indicate ^T^ the type strain of a species and ^P^ the pathotype strain for a pathovar*.

*Superscript asterisk following strain name indicates strains with a suspected misnaming based on core genome phylogeny. Culture collections providing strains are abbreviated in the strain names as ATCC (American Type Culture Collection, Manassas, Virginia, United States), CFBP (Collection Française de Bactéries associées aux Plantes, FR), DSM (German Collection of Microorganisms and Cell Cultures, DE), ICMP (International Collection of Microorganisms from Plants, NZ), LMG (Bacteria collection of the Laboratory for Microbiology of the Faculty of Sciences of the Ghent University, BE), NCPPB (National Collection of Plant Pathogenic Bacteria, United Kingdom), and MAFF (NIAS Genebank of the Ministry of Agriculture, Forestry and Fisheries, JP)*.

2 *N.D.: not determined*.

3 *For Whole Genome Shotgun (WGS) sequences, accession numbers are provided as four letters prefixes and two digits for the version number of the data set*.

4 *N.A.: not applicable*.

The core genome phylogenetic relationships were obtained using EDGAR 2.2 (Blom et al., 2016) as previously described (Ruinelli et al., 2019).

### Comparative Genomics, Gene Sets Calculation, and Identification of *Prunus*-Associated Genes

**Figure 1:**
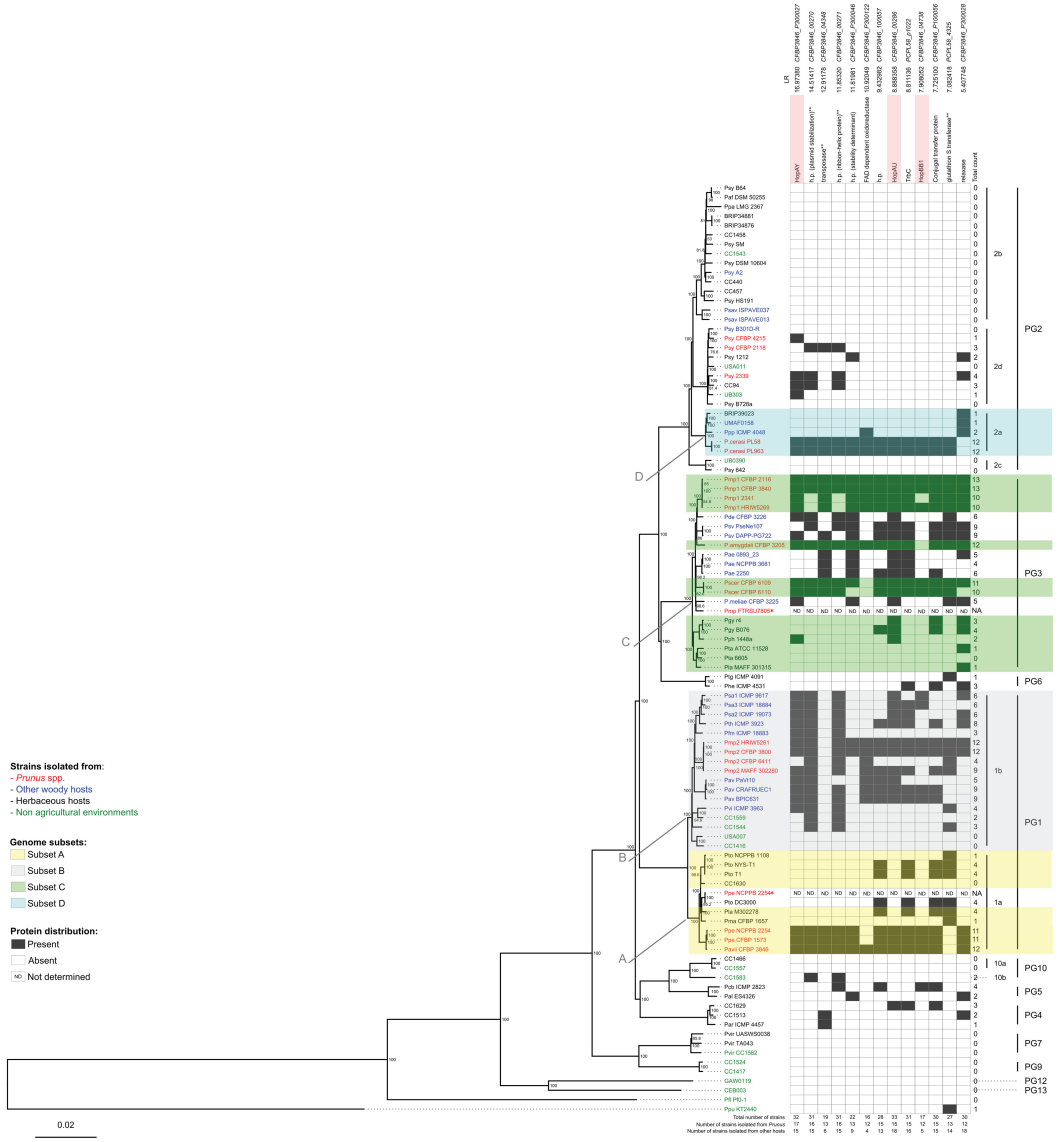
Neighbor-joining (NJ) phylogeny based on the core genome of the *Pseudomonas syringae* species complex and distribution profile of 13 proteins considered to be significantly associated with *Prunus* spp. among these strains. A set of 99 genomes of strains belonging to the *P. syringae* species complex as well as one *Pseudomonas fluorescens* and one *Pseudomonas putida* strains were used for this analysis (Table 1). The tree was built using EDGAR 2.2 (Blom et al., 2016) out of a core genome of 1,344 CDS giving a total alignment of 5,36,722 amino acids per genome. Percent bootstrap (bt) support values calculated for 500 reiterations are indicated near nodes. Only bt values over 51 are displayed. The strain names refer to the code field from Table 1. Phylogroups (PG) and clades are indicated on the right. Strains isolated from *Prunus* spp. are indicated in red, from other woody hosts in blue, from herbaceous hosts in black, and from non-agricultural environments in green. Strain names followed by an asterisk (*) indicate strains which were excluded from further comparative analyses due to a presumed misnaming of the genome. Genome subsets (A-D) used to determine the correlation between gene presence and *Prunus* spp. association are indicated with color highlights. Arrows indicate the node at which *Prunus*-associated strains are diverging from the non-*Prunus*-associated strains within the same genome subset. Protein orthologs were retrieved out of these 97 genomes using EDGAR 2.2 (Blom et al., 2016). Black squares indicate presence of the protein based on FIGURE 1the orthology criteria of EDGAR 2.2. Proteins highlighted in pink are involved in virulence based on their annotation. Protein descriptions followed by two asterisks (**) indicate that orthologs were also found using online TBLASTN analysis against 13 additional *Pseudomonas* species closely related to the *P. syringae* species complex as reported in Supplementary Figure 4. The proteins are ordered by decreasing significance of the likelihood ratio (LR) statistic when exceeding the *p* ≤ 0.05 threshold of 5.36. This order is not indicative of any physical proximity. Locus tags and LR statistic are reported over each considered protein; h.p.: hypothetical protein; NA: not applicable; and ND: not determined.

Based on the core genome phylogeny, four subsets of genomes were defined (subsets A–D; Table 1) to be used in comparative genomics. Within each of the subsets, the sets of orthologous proteins present in *Prunus*-associated strains but absent in their phylogenetically closely related non-*Prunus*-associated strains were determined using EDGAR 2.2 (Blom et al., 2016). The protein sequences (*n* = 1,058) resulting for each of the subsets (subsets A–D; Figure 1) were used as large query against each other using standalone BLAST v.2.2.29+ (Camacho et al., 2009). All BLASTP hits having identity and coverage higher or equal to 70% were considered as ortholog and displayed in a Venn diagram. Orthologous proteins shared among each combination of subsets (*n* = 52) were checked for orthologs in the whole set of genomes (*n* = 97) using EDGAR 2.2.

Using the core genome phylogeny as a reference, associations were identified between the presence/absence of each orthologous protein in the analyzed genomes (*n* = 99) and the discrete binary trait designated “*Prunus* spp. isolate” or “other host/environment isolate” using BayesTraits v.3.0.5 (Pagel, 1994; Barker and Pagel, 2005; Pagel and Meade, 2006). The goodness of fit of the dependent versus independent model was compared with a likelihood ratio (LR) test by using a Perl script to run both models (available from https://github.com/reubwn/bayestraits-wrapper; Nowell et al., 2016). The LR test was conducted for the 52 genes that occurred in either greater than six or fewer than 92 strains, resulting in a total of 49 LRs. A null LR distribution model was constructed by randomly permuting a total of 100 times either the gene occurrence data for each of the 52 tested genes, the binary trait designation or both variables, in each case calculating a new LR statistic (Nowell et al., 2016). The null distribution was then used to derive the *p*-value thresholds. The proteins considered to be significantly more present in *Prunus*-associated strains were also used as online TBLASTN queries against nucleotide databases from 13 additional *Pseudomonas* species closely related to *P. syringae* (Mulet et al., 2010; Lalucat et al., 2020; Supplementary Figure 4). The presence of an ortholog in any of these 13 closely related *Pseudomonas* species was then reported when at least one TBLASTN hit having identity and coverage higher or equal to 70% was detected.

### HopAY and HopAR Ortholog Retrieval and Phylogenetic Analysis

The bidirectional best hits protein orthology criteria used in EDGAR 2.2 in the previous step is mostly designed to determine the presence of a complete and probably functional ortholog protein among different genomes. However, in order, to investigate the evolution of a gene within different strains it is also important to differentiate between absence or inactivation of that gene. For this purpose, the *hopAY* reference sequence (GenBank accession number CP000059.1; locus tag: PSPPH_A0129) was derived from the T3E database (PPI, 2010) and used as online BLASTN query against all genomes selected for comparative genomics (*n* = 97). The resulting nucleotide sequence was translated using the ExPASy translate tool (ExPASy) and the longest open reading frame corresponding to the reference HopAY sequence (GenBank accession number: AAZ37994.1) was used for alignment. Deviations between the BLASTN hit and the identified protein were investigated in comparison to the reference *hopAY* gene for the possibility of pseudogenization due to frameshift or insertion of a stop codon in the correct reading frame. DNA and amino acid sequences were aligned using ClustalW, while MEGA 6.0 was used to generate neighbor-joining (NJ) phylogeny using the Jones–Taylor–Thornton model with the gamma parameter set at 2.25 and bootstrap values after 1,000 repeats as suggested elsewhere (Lindeberg et al., 2005). A similar method was used twith *hopAR* (GenBank accession number AJ870974.1 positions 17,471–18,274) and *hopAU* (GenBank accession number LT963409.1; locus tag: CFBP3840_01698).

### Comparison of the *Arabidopsis* PBS Resistance Protein Among Different Plant Species

The T3E HopAR1 (formerly AvrPphB) from *P. syringae* pv. phaseolicola belongs to the same family of C58 protease as HopAY and has been shown to proteolytically cleave the serine/threonine protein kinase PBS1 in *Arabidopsis*. The amino acid sequence of PBS1 from *Prunus persica* (GenBank accession number XP_007225732) was used to perform a TBLASTN search in the Transcriptome Shotgun Assembly Sequence (TSA) and Protein NCBI databases of the plants associated with strains possessing a full-length or truncated HopAY.

The transcribed mRNA sequences retrieved from the TSA were translated using the ExPASy translate tool and the obtained amino acid sequences were aligned to the PBS1 amino acid sequence retrieved from the NCBI protein database of 22 additional plant species (Supplementary Table 1) using ClustalW on the MEGA 6.0 software. To clarify the phylogenetic relationships among the PBS1 proteins of different plants, a maximum likelihood phylogeny was reconstructed using the Jones–Taylor–Thornton model with the gamma parameter set at 2.25 and bootstrap values of 1,000.

### Data Availability

The data sets analyzed for this study are available at the NCBI GenBank/DDJ/EMBL database under the accession detailed in Table 1.

## Results

### Phylogenomics

In order to clarify the exact phylogenetic position of the *Prunus*-associated strains in the data set within the *P. syringae* species complex and to define suitable strains and subgroups for comparative genomics (Table 1), a core genome-based phylogeny was generated for the selected set of genomes using EDGAR 2.2 (Blom et al., 2016). The obtained tree was generated based on the concatenated and aligned amino acid sequences of 1,344 genes consisting of a total length of 536,722 amino acids (Figure 1).

The main clustering obtained from the core genome phylogeny reflects the PG previously defined by Multi Locus Sequence Analysis (MLSA; Sarkar and Guttman, 2004; Hwang et al., 2005; Sarkar et al., 2006) and single locus phylogeny (Parkinson et al., 2011; Berge et al., 2014). However, our analysis revealed that two genomes obtained from the Whole Genome Shotgun (WGS) NCBI database which were supposed to represent strains isolated from *Prunus* spp. did not cluster as expected based on previous work (Parkinson et al., 2011). Indeed, the sequence with the GenBank WGS accession prefix LAZV01 which is supposed to represent *P. syringae* pv. persicae strain NCPPB 2254 and should cluster close to *P. syringae* pv. avii (Parkinson et al., 2011) was found to be clustering really close to the complete genome of *P. syringae* pv. tomato DC3000 and quite distant from the two other *P. syringae* pv. persicae genomes generated previously (Ruinelli et al., 2019). Additionally, the sequence with accession number LGLQ01 which was deposited in the NCBI database as *P. amygdali* pv. morsprunorum strain FTRSU7805 clustered closer to *P. syringae* pv. cerasicola and *Pseudomonas meliae* than to other strains of *P. syringae* pv. morsprunorum race 1. This observation was supported by the calculation of the average nucleotide identity (ANI) values among the suspected strains, their observed phylogenetically closely related strains and their supposed closely related strains (Supplementary Figure 1). Considering these facts, the sequences with the WGS accession prefixes LAZV01 and LGLQ01 were not included in further comparative genomics analysis.

### Correlation Between Genes Presence and *Prunus* spp. Association

**Figure 2:**
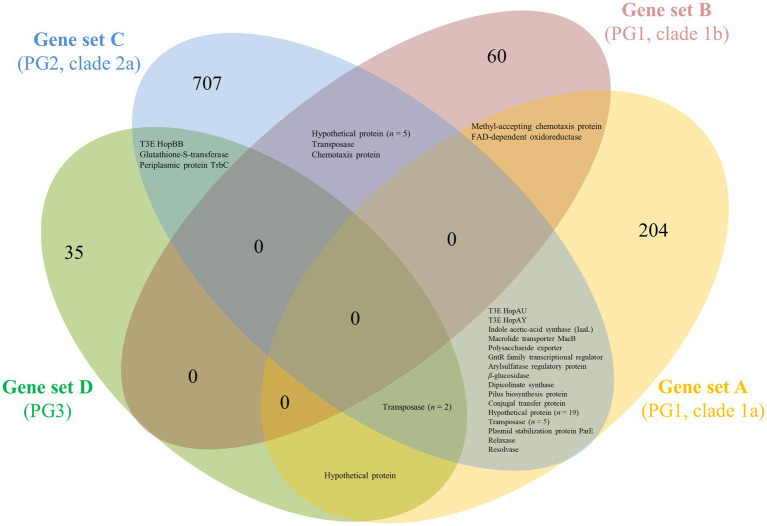
Venn diagram showing groups of ortholog proteins within *Prunus*-associated *Pseudomonas syringae* strains belonging to different PG but absent from phylogenetically closely related non-*Prunus*-associated strains. Subsets of genomes to be compared were defined based on core genome phylogeny within PG1a (subset A), PG1b (subset B), PG2a (subset C), and PG3 (subset D; see Figure 1). For each subset of genomes, the core genome of the *Prunus*-associated strains was calculated, and at the same time, all ortholog proteins found in non-*Prunus*-associated strains within the same subset were discarded using EDGAR 2.2 (Blom et al., 2016). The protein sequences resulting for each of the subsets (gene sets A–D) were used as BLASTP query against each other using standalone BLAST v.2.2.29+ (Camacho et al., 2009) and considered as ortholog if identity and coverage were higher or equal to 60%.

The number of orthologous proteins present in *Prunus*-associated strains but absent in non-*Prunus*-associated strains retrieved for each of the compared genome subsets (subsets A–D; Table 1; Figure 1) ranged from 41 (PG3, genome subset D) to 758 (PG2a, genome subset C; Figure 2). This considerable difference could be because *Prunus*-associated strains within PG3 belonged to different pathovars and species (*P. syringae* pv. cerasicola, *P. syringae* pv. morsprunorum race 1 and *P. amygdali*), whereas within PG2a only strains of *P. cerasi* have been described to date as being associated with *Prunus* diseases. Among *P. syringae* pv. avii and *P. syringae* pv. persicae (PG1a, genome subset A), a relatively high number of orthologous proteins were retrieved (*n* = 249), whereas only 70 orthologous proteins were found within strains of the genome subset B (PG1b; Figure 2).

To verify which proteins were shared among *Prunus* spp. associated strains belonging to different PG, the proteins retrieved for each genome subset (*n* = 1,058) were compared for orthology using BLASTP and the results obtained for each possible combination represented in a Venn diagram (Figure 2). A total of 52 proteins were found to be shared at least between two genome subsets but no protein was found to be shared among all *Prunus*-associated members of the *P. syringae* species complex. Each protein was checked for distribution across all the initially selected set of genomes (*n* = 97). None of the analyzed proteins (*n* = 52) was found exclusively in *Prunus*-associated strains but 19 of them were found to be significantly more abundant in *Prunus*-associated strains than in non-*Prunus*-associated strains (likelihood ratio statistic exceeding the *p* ≤ 0.05 threshold of 5.36; Figure 1; Table 2; Supplementary Figure 2). Out of these, only proteins present in at least 60% of the *Prunus* spp. isolated strains were finally considered, giving a total of 13 proteins (Figure 1; Table 2). Strains isolated from *Prunus* spp. belonging to PG1a, PG1b, PG2a, and PG3 possessed a similar distribution profile with exception of the *P. syringae* pv. morsprunorum race 2 strain CFBP 6411 (PG1b) and strains from PG2d which were more divergent (Figure 1).

**Table 2:** Number of genes significantly more present in *Prunus* spp. isolated strains.

*p*-value	LR value	Number of genes			Proportion (%)	
		Expected^1^	Observed	Retained^2^	Tested^3^	Flexible^4^
0.05	5.36	3	19	13	38.78	1.80
0.01	6.40	<1	17	12	34.69	1.61
0.001	9.08	<1	11	7	22.45	1.04
0.0001	12.62	<1	3	3	6.12	0.28
0.00001	12.63	<1	3	3	6.12	0.28

1 *Expected number of Type I (false-positive) errors under the null model*.

2 *Retained based on the criteria present in 60% of the Prunus isolated strains*.

3 *Proportion of the 49 tested genes (three genes skipped based on the criteria occurring in either greater than six or fewer than 92 strains)*.

4 *Proportion of the total flexible genome of subsets A-D (1,058 genes)*.

A third of the analyzed proteins were hypothetical proteins (*n* = 4) and also a third were located potentially on plasmids (*n* = 4) when complete genomes were available (Table 1; Supplementary Figure 3). However, three known virulence factors were found to be significantly more present in *Prunus* spp. associated members of the *P. syringae* species complex, namely, three T3E (HopAY, HopAU, and HopBB; Figure 1). These three known virulence factors were only reported in the species *P. syringae* during ortholog analysis within 13 additional *Pseudomonas* species closely related to the *P. syringae* species complex (Supplementary Figure 4).

The T3E HopAY was the protein with the highest LR statistic and the most abundant in *Prunus* spp. associated strains (89%) if compared to all other considered proteins (*n* = 12) and it was found only in 19% of strains isolated from other hosts or from non-agricultural environment (Figure 1). Within strains of the PG2 (*n* = 31) only six strains harbored HopAY of which four were isolated from *Prunus* spp. (Figure 1).

**Figure 3:**
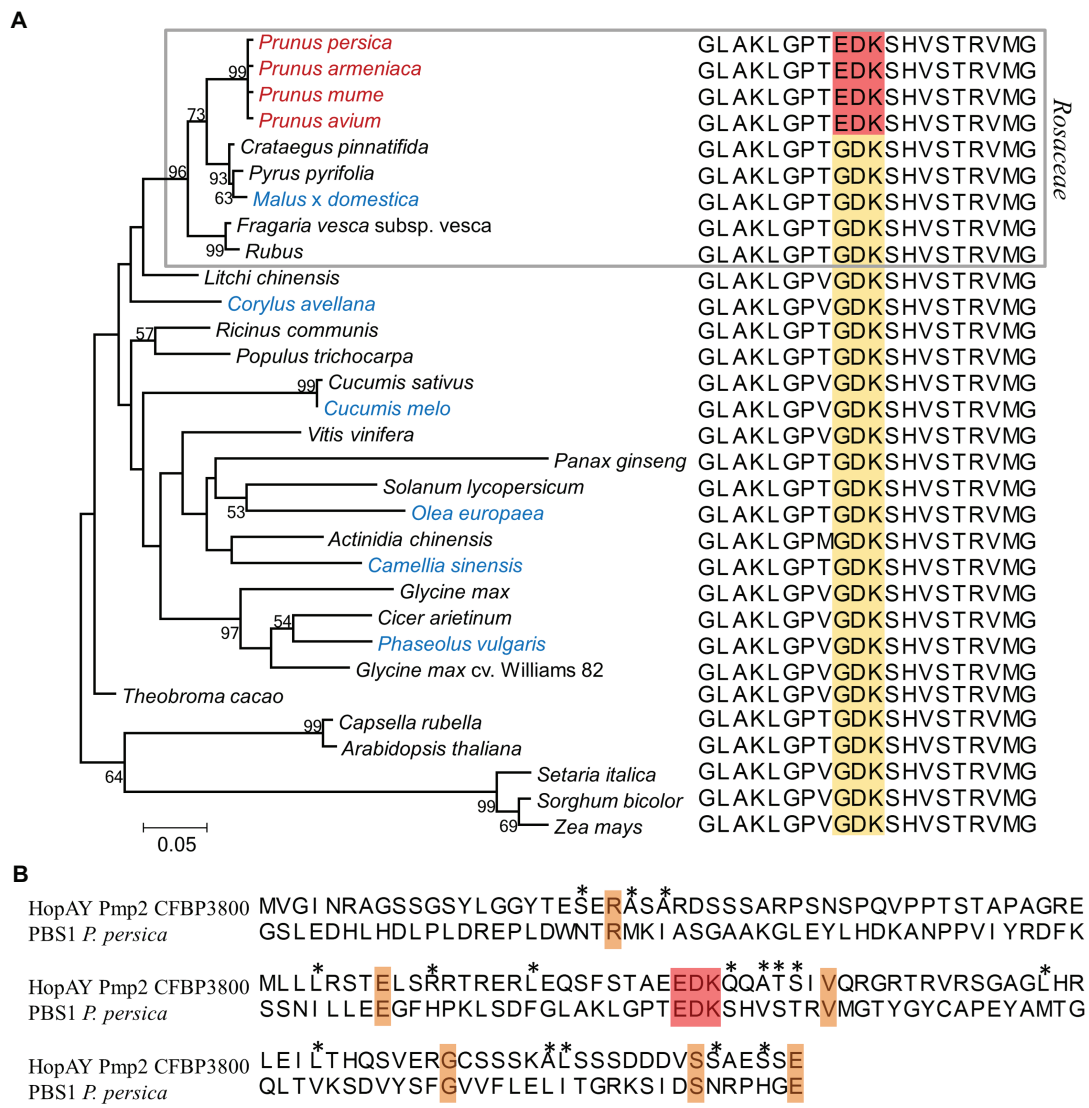
Comparison of the serine/threonine protein kinase PBS1 among different plant species **(A)** and relation to the HopAY sequence **(B)**. **(A)** Maximum likelihood phylogeny of the PBS1 protein among different plant species and relative PBS1 sequence stretch corresponding to the amino acids 233–252 of the *Arabidopsis thaliana* PBS1 sequence (NCBI Acc. Nr. NP_196820) containing the HopAR1 cleavage site GDK (brown block). The corresponding EDK region in the PBS1 sequence of the *Prunus* spp. is highlighted in red. In the phylogeny *Prunus* spp. members are reported in red, whereas plants other than *Prunus* spp. from which *Pseudomonas syringae* strains possessing the HopAY gene were isolated are indicated in blue. The gray block indicates species belonging to the *Rosaceae* family. The evolutionary distances were computed using the Jones–Taylor–Thornton model matrix-based with a gamma distribution (shape parameter = 2.25). Percent bootstrap (bt) support values calculated for 1,000 reiterations are indicated near node. Only bt values over 51 are displayed. All ambiguous positions were removed for each sequence pair giving a total of 598 positions. Alignments were obtained using ClustalW and phylogenetic analysis was performed with MEGA 6.0. **(B)** Alignment of HopAY and PBS1 around the EDK region. The first 133 amino acids (aa) of the HopAY sequence of *Pseudomonas syringae* pv. morsprunorum race 2 CFBP 3800 (Pmp2 CFBP3800) were aligned to the aa 170–302 of the *Prunus persica* PBS1 sequence (NCBI Acc. Nr. XP_007225732). The EDK motif is highlighted in red, identical residues are highlighted in orange, whereas residues sharing similar side chain properties at a specific position are indicated by asterisks.

A similar distribution was observed for the T3E HopAU, which was present in 80% of *Prunus*-associated strains and 23% of strains isolated from other hosts. Out of the 32 strains possessing HopAY, 27 also possessed HopAU (Figure 1; Supplementary Figure 5). The T3E HopBB was present in only 6% of non-*Prunus* isolated strains but its abundance was also lower in strains isolated from *Prunus* (63%, Figure 3).

HopAY is predicted to belong to the same class of C58 peptidases like the well-characterized T3E HopAR (formerly AvrPphB). HopBB has been shown to interact with regulators of the jasmonic acid hormone signaling pathway in *Arabidopsis* (Yang et al., 2017), whereas HopAU was recently shown to activate plant immunity by interacting with a calcium-sensing receptor in *Nicotiana benthamiana* and in kiwifruit (Zhang et al., 2022).

### Sequence Comparison of HopAY and HopAR, a Very Well-Characterized C58 Cysteine Protease in *Pseudomonas syringae*

The T3E HopAY showed the strongest level association with *Prunus* isolated strains in respect to all other genes (*n* = 13) analyzed in this study (Figure 1) and belongs to the C58 cysteine proteases family. Another well-studied and characterized T3E encoding for a C58 peptidase is HopAR which was initially identified in *P. syringae* pv. phaseolicola as being responsible for elicitation of HR in bean (Jenner et al., 1991; Puri et al., 1997). Orthologs of *hopAR* were retrieved from 10 out of 19 strains isolated from *Prunus* spp. and in 13 strains isolated from other hosts (Supplementary Figure 5). Around 15 strains, including nine strains isolated from *Prunus* spp., possessed both *hopAY* and *hopAR* orthologs. The target of HopAR in *Arabidopsis* is the serine/threonine protein kinase AVRPPHB SUSCEPTIBLE 1 (PBS1) and the ability of HopAR to cleave PBS1 is related to the presence in PBS1 of the Glycine (G241)-Aspartate (D242)-Lysine (K243) motif which is also found at the autocleavage site of HopAR (Shao et al., 2003). Mutations in the amino acids G241, D242, and K243 of PBS1 in *Arabidopsis* reduced the proteolytic activity of HopAR by 90, 75, and 15%, respectively (Shao et al., 2003). The cleavage of PBS1 by HopAR induces a conformational change of PBS1 causing the exposition of a particular motif (SEMPH) which is sensed by the resistance protein RESISTANCE TO PSEUDOMONAS SYRINGAE 5 (RPS5) in *Arabidopsis*, leading to HR (Ade et al., 2007; Qi et al., 2012, 2014). In addition, the determination of the crystal structure of HopAR1 revealed the presence of a catalytic triad composed by a cysteine (C98), histidine (H212), and aspartate (D227) which has been shown to be essential for catalysis (Zhu et al., 2004). As already noticed by Zumaquero et al. (2010), the amino acid sequence similarity between HopAY and HopAR is very limited (68% query coverage and 27% identity; Supplementary Figure 6). Nevertheless, motifs corresponding to the catalytic triad were identified also on HopAY and localized at C156, H265, and D280 using the HopAY reference present in the T3E database (PPI, 2010; NCBI locus tag: PSPPH_A0129), whereas no motif corresponding to the cleavage site of HopAR (GDK) was found in the HopAY sequence (Figure 3B). Secondary structure prediction revealed a conserved pattern of α-helices and β-sheets between HopAR and HopAY as well as other members of the C58 proteases (Zhu et al., 2004). Alignment of the PBS1 protein sequence from different plant species (*n* = 31) revealed that the protein kinase PBS1 is quite conserved among different plant families (Qi et al., 2014). However, we noticed that members of the *Prunus* spp. (*n* = 4) possess an EDK motif instead of the GDK motif essential for HopAR cleavage in PBS1, which was in contrast conserved in all other plant species included in the comparison (*n* = 27; Figure 3A). The alignment of the PBS1 sequence of *P. persica* with HopAY revealed that the same EDK motif was found also within the N-terminal half of HopAY (E76, D77, and K78) followed by a stretch of four amino acids with the same physical properties (Figure 3B). In addition, all PBS1 sequences analyzed in this study with exception of PBS1 of *Arabidopsis thaliana* and *Capsella rubella* were also lacking the SEMPH motif, which was shown to be essential for RPS5 mediated resistance in *Arabidopsis* (Qi et al., 2014).

### Sequence Comparison of HopAY Among Different Members of the *Pseudomonas syringae* Species Complex

In order to determine the evolutionary relationships of *hopAY* within different strains of the *P. syringae* species complex, a BLASTN search was performed using the *hopAY* sequence of *P. syringae* pv. phaseolicola 1448a (PPI; NCBI locus tag: PSPPH_A0129) against the set of genomes selected for comparative genomics (*n* = 97; Table 1). The BLASTN analysis revealed the presence of 43 *hopAY* sequences in a total of 36 strains. In addition to the strains retrieved by the protein-based ortholog search (*n* = 32; Figure 1), a *hopAY* ortholog was present in the horse chestnut-associated *P. amygdali* pv. aesculi strains 2250, 0893_23, and NCPPB 3681 as well as in the apple tree pathogen *P. syringae* pv. papulans ICMP 4048. With exception of strain HRIW5269, all other *P. syringae* pv. morsprunorum race 1 strains analyzed in this study (*n* = 3) were possessing more than one copy of *hopAY*. In the genomes of *P. syringae* pv. avii strain CFBP 3846, *P. amygdali* CFBP 3205, and *P. syringae* pv. dendropanacis CFBP 3226, two copies of *hopAY* were found as well.

**Figure 4:**
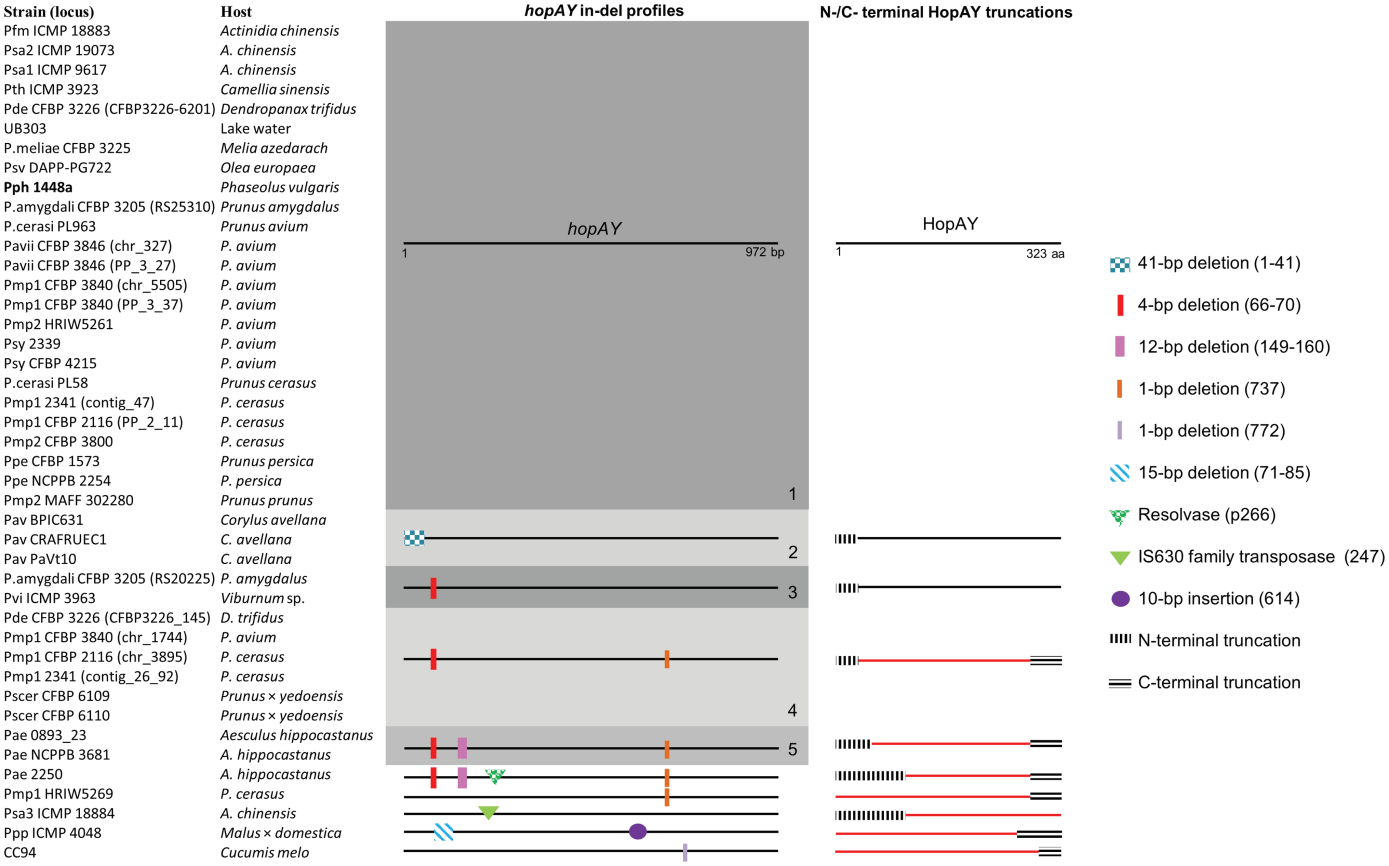
Insertion–deletion profiles of *hopAY* retrieved by BLASTN and corresponding HopAY truncation scheme. Five major groups (1–5) were defined based on conserved indel mutations. Numbers in bracket indicate the position of the insertion or deletion based on the reference *hopAY* sequence from *Pseudomonas syringae* pv. *phaseolicola* 1448a (indicated in bold) available in the Hop database (PPI). HopAY proteins with a truncation affecting the catalytic domains characteristic for C58 cysteine protease and thus considered as non-functional are represented in red. The strain names used refer to the code field from Table 1. Figure is not to scale.

Sequence analysis revealed that the retrieved *hopAY* sequences (*n* = 43) could be divided into five major groups based on the insertion–deletion (indel) scheme affecting this gene (Figure 4). The indel group 1 (*n* = 25) consisted of sequences with no insertions or deletions if compared to the reference *hopAY* sequence available in the *hop* database and were mostly retrieved from genomes of strains isolated from *Prunus* spp. (*n* = 16). Sequences belonging to the indel group 2 (*n* = 3) were affected by a probable transposase insertion leading to a 41-bp deletion at the 5′ end (Figure 4) and were retrieved only from *Pseudomonas avellanae* strains. Indel groups 3, 4, and 5 displayed an additive indel profile. In fact, the indel group 3 (*n* = 2) displayed a 4-bp deletion at position 66–70 which was shared also from groups 4 (*n* = 6) and 5 (*n* = 2). A 1-bp deletion located at position 737 was also present in sequences of groups 4 and 5, whereas group 5 was additionally having a 12-bp deletion at position 149–160. Sequences of the indel group 4 were retrieved only from strains of the PG3 and mostly isolated from *Prunus* spp. (*n* = 5), namely, *P. syringae* pv. cerasicola and *P. syringae* pv. morsprunorum race 1. Based on the complete genomes previously sequenced using PacBio (Ruinelli et al., 2019), it was possible to determine that all *hopAY* of the indel group 4 were located on the chromosome, whereas the *hopAY* of indel group 1 were located on both chromosome and plasmids. Sequences of the indel group 5 were retrieved from two *P. syringae* pv. aesculi strains isolated in Europe, whereas the *P. syringae* pv. aesculi isolated in India displayed an additional resolvase insertion within *hopAY* (Figure 4). In addition to the above-described groups, four sequences displayed unique indel profiles varying from transposase insertions (Psa ICMP 18884) to 1-bp deletions (CC94; Figure 4).

Alignment of HopAY sequences retrieved from the BLASTN search (*n* = 43) revealed that sequences belonging to the previously described indel groups 4 and 5 as well as four of five additional sequences with unique indel profiles (Figure 4) were missing both H256 and D280 due to the introduction of a premature stop codon (Figure 4). On the other side, the transposase insertion within *hopAY* of *P. syringae* pv. actinidiae ICMP 18884 led to a N-terminal truncation deleting the C156 motif. In addition, the HopAY from *P. amygdali* CFBP 3205 belonging to the indel group 1 possessed a tyrosine instead of the expected H256. With exception of both *P. syringae* pv. cerasicola strains, the other three strains isolated from *Prunus* belonging to the indel group 4 thus possess an inactivated HopAY and at least another copy of *hopAY* encoding a full-length protein. The N-terminal truncations observed in the *P. avellanae* strains (indel group 2) and in the sequences of the indel group 3 did not affect the catalytic triad of HopAY and thus it was not possible to determine if the derived protein would be functional or not (Figure 4).

### Phylogeny of HopAY

**Figure 5:**
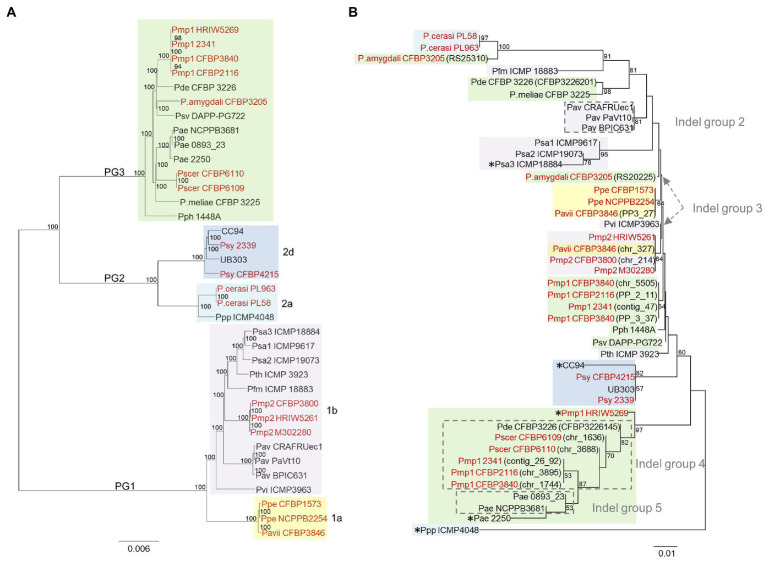
Comparison of the neighbor-joining phylogeny based on the core genome **(A)** and on HopAY **(B)**. **(A)** The core genome of the 36 strains possessing an *hopAY* ortholog based on the BLASTN search was determined using EDGAR 2.2 (Blom et al., 2016) out of a core genome of 2,511 CDS giving a total alignment of 872,675 amino acids per genome. The strain names used refer to the code field from Table 1. Phylogroups and clades are indicated on the left and on the right, respectively. **(B)** The phylogeny of the 43 retrieved HopAY sequences was computed using the Jones–Taylor–Thornton model matrix-based with a gamma distribution (shape parameter = 2.25). Percent bootstrap (bt) support values calculated for 1,000 reiterations are indicated near nodes. Only bt values over 51 are displayed. All ambiguous positions were removed for each sequence pair giving a total of 323 positions in the final data set. Evolutionary analyses were conducted in MEGA 6.0. Indel groups (gray dashed lines boxes) refer to Figure 4. Asterisks (*) indicate strains with unique indel profiles. If nothing stated, sequences belong to indel group 1. For strains possessing multiple copies of HopAY, the locus tag is indicated in brackets. Strains isolated from *Prunus* spp. are highlighted in red. PG and clades are indicated with the same color code as used on the left in panel **(A)**.

The NJ phylogeny obtained from the 43 retrieved HopAY sequences did not reflect the phylogeny obtained from the core genome of the 36 strains possessing a *hopAY* ortholog (Figures 5A,B). In particular, the HopAY sequence of *Prunus*-associated strains belonging to PG1a, PG1b, PG2a, and PG3 cluster closer to each other than to strains isolated from other hosts belonging to the same PG.

For example, HopAY sequences from *P. syringae* pv. morsprunorum race 2 strains belonging to clade PG1b cluster closer to *Prunus* isolated strains of PG1a than to strains of the PG1b, namely, *P. syringae* pv. actinidiae and *P. avellanae* (Figure 5B). In addition, protein sequences from strains of the PG2d form a monophyletic cluster, which is distantly related to strains of the PG2a clade (Figure 5B). Within PG2a, the proteins from *Prunus*-associated *P. cerasi* strains are more closely related to the full-length protein of the PG3 strain *P. amygdali* CFBP 3205, also isolated from *Prunus*, than to that of *P. syringae* pv. papulans strain ICMP 4048 (PG2a). HopAY sequences belonging to the indel groups 4 and 5, which were all retrieved from members of the PG3, form a clearly separated cluster together with the proteins from two strains with unique indel profile, being *P. syringae* pv. morsprunorum race 1 HRIW5269 and *P. syringae* pv. aesculi strain 2250 (Figure 5B).

## Discussion

The development of effective measures to control plant diseases would be facilitated by a founded knowledge on the pathogen biology as well as on mechanisms involved in the plant–pathogen interactions. Diseases caused by members of the *P. syringae* species complex on species belonging to the *Prunus* genus are responsible for relevant yield losses, particularly in young orchards (Puławska et al., 2017). To date, a total of two species and six pathovars belonging to three different PG of the *P. syringae* species complex have been found in association with diseases of species within the *Prunus* genus. Despite their economic importance, not much is known about the evolution and adaptation strategies of members belonging to the *P. syringae* species complex toward *Prunus* spp. In this study, a whole-genome comparison approach was used aiming to identify genetic traits shared among these phylogenetically distantly related pathovars and species that could give insights into the evolutionary aspects related to the adaptation toward *Prunus* spp. hosts.

From the core genome-based phylogeny obtained in this study, it was evident that the pathoadaptation toward *Prunus* spp. is not the result of a single evolutionary event but have evolved independently at least three times in the evolutionary history of the *P. syringae* species complex. This convergent pathoadaptation in distantly related strains leading to virulence on the same host is not unique for the *P. syringae*—*Prunus* spp. pathosystem within the *P. syringae* species complex (Morris et al., 2019). In fact, phylogenetically distantly related members of the *P. syringae* species complex were also found to have converged onto hazelnut (Wang et al., 2007).

Wang et al. (2007) explained the occurrence of convergent pathoadaptation toward a specific host is not only by the independent acquisition of genes necessary for a successful association but also by the specific loss or inactivation of genes resulting in the same host range limitation. The predominant evolutionary force driving such events in the *P. syringae* species complex is horizontal gene transfer (HGT), which allows the transfer (gain or loss) of genes between closely and distantly related strains within relatively short evolutionary periods (Nowell et al., 2014). Based on HGT, genes having a selective advantage can be easily accumulated leading to new pathovars or lineages which can adapt to new ecological niches and hosts.

The comparative genomic analysis performed in this study revealed a strong correlation between the presence of the T3E HopAY and the association of members of the *P. syringae* species complex with hosts belonging to the genus *Prunus*. The gene *hopAY* was claimed to be significantly associated with the woody host niche (Nowell et al., 2016), something that we also noticed if considering the *hopAY* orthologs. However, our analysis highlighted the importance of considering not only the gene sequence but also the protein sequence to correctly interpret T3E profiles. A few studies recently took this also into consideration and showed that T3E alleles were linked to host specificity (Zembek et al., 2018; Jayaraman et al., 2020).

Unlike many T3E which have no known function, HopAY is a putative member of the C58 cysteine protease family which is characterized by the presence of an invariant catalytic triad composed by a Cysteine (C), a Histidine (H), and an Aspartate (D) which are essential for catalysis (Shao et al., 2002). Based on that knowledge, it was possible to determine that half of the *hopAY* sequences retrieved based on DNA orthology were encoding for proteins missing at least one of those essential amino acids (C/H/D) and thus would not be functional. Inactivated HopAY were found also in *Prunus*-associated strains but most of them were shown to possess an additional *hopAY* encoding for a full-length protein possessing the C/H/D catalytic triad, often located on a plasmid. The evolutionary dynamics observed within the retrieved HopAY sequences suggests that this protein may be of selective disadvantage on certain hosts and therefore mutated at higher rate than other T3E, like already observed for other T3E families (Baltrus et al., 2011). The phylogeny obtained based on HopAY did not reflect the core genome-based phylogeny, thus excluding a vertical pattern of inheritance and further support the importance of HGT as adaptive force in the evolution of the *P. syringae* species complex. In addition, it revealed that the HopAY sequence present in many *Prunus* spp. associated strains belonging to PG1a, PG1b, and PG3 was nearly identical, supporting the theory of convergent pathoadaptation of these strains.

HopAR (former AvrPphB), another T3E of the C58 cysteine protease family, was subject of many molecular studies in the last decades. These studies revealed that HopAR targets the protein kinase PBS1 in *Arabidopsis* due to the presence of a particular recognition motif (GDK) which was also found in the sequence of HopAR (Shao et al., 2003). Cleavage of PBS1 by HopAR could result in increased virulence or lead to resistance in *Arabidopsis* plants lacking or possessing the resistance protein RPS5, respectively (Ade et al., 2007). The PBS1 protein is quite conserved among different plant species representing a good target for T3Es. In contrast to all other considered plant species, including other members of the *Rosaceae* family, the PBS1 sequence found in *Prunus* spp. lacked the GDK motif necessary for HopAR cleavage and possessed instead an EDK motif which was found also in the N-terminal half of HopAY. The N-terminal part of members of the C58 cysteine protease family is known to be involved in substrate specificity as shown for HopAR and for the DKM motif of Y4zC, a putative T3E of *Rhizobium* (Zhu et al., 2004). Based on this observation, we speculate that HopAY could act in a similar way as HopAR but specifically evolved to cleave the PBS1 ortholog of *Prunus* spp., thus explaining why HopAY is significantly associated with strains adapted to this group of hosts. This hypothesis is supported by the fact that strains isolated from other hosts, such as *Corylus avellana* and *Aesculus hippocastanum*, both harboring a GDK motif in the PBS1 sequence, possessed a truncated or non-functional HopAY, respectively. In addition, Zumaquero et al. (2010) showed that knocking out HopAY does not affect pathogenicity of *P. syringae* pv. phaseolicola 1448a on bean, whose PBS1 protein also possesses a GDK motif. Of course, it could also be hypothesized that the PBS1 protein in *Prunus* has evolved to be cleaved by HopAY to trigger resistance by action of a third unknown resistance protein (similarly to RPS5). However, pathogenicity tests using wild-type strains revealed no direct correlation with presence or absence of *hopAY* (Ruinelli et al., 2019). Therefore, this suggests that *Prunus* spp. does not possess a recognition system for HopAY. At the same time, based on that data, HopAY does not seem to be the determinant factor for pathogenicity but it could still play a role interfering with plant immune response. In order to confirm this hypothesis, additional experiments are needed to show that HopAY is a functional protease able to cleave PBS1 from *Prunus* spp. but the comparative genomic analysis conducted here already provided evidence for sequence correlation between HopAY and its putative cognate target in *Prunus* spp.

This study identifies traits supporting the adaptation between members of the *P. syringae* species complex with species belonging to the *Prunus* genus. It also revealed that most of the mutations affecting *hopAY* were short insertions or deletions that would not be detected by regular PCR and gel electrophoresis, a method that was often used to determine T3E profiles of *P. syringae* and other plant pathogens before the advent of next-generation sequencing technologies (Escalon et al., 2013; Ferrante and Scortichini, 2015). Besides highlighting the biases linked to DNA-based T3E profiling, this study also underlines the importance of integrating host genomic data to correctly interpret the relevance of genomic traits found in the pathogen.

## Data Availability Statement

The original contributions presented in the study are included in the article/**Supplementary Material**, further inquiries can be directed to the corresponding author.

## Author Contributions

TS and JP conceptualized the study with the assistance of MR. MR designed the methodology and carried out the experiments. MR and JP analyzed the data with the assistance from JB and TS, contributed to the data visualization, prepared the original draft with assistance from JB and TS for review and editing, and curated the data. JB and JP helped with software. All the authors revised the final version of the manuscript, while JP acted as the corresponding author. All authors contributed to the article and approved the submitted version.

## Funding

This research was funded by the Swiss Secretariat for Education, Research and Innovation, grant number SBFI C12.0099, within the European research network COST Action FA1104 “Sustainable production of high-quality cherries for the European market” and in part by the European Union Seventh Framework (FP7/2007–2013) under the grant agreement no. 613678 (DROPSA). TS and JP were supported by the Department of Life Sciences and Facility Management of ZHAW. The EDGAR platform is financially supported by the BMF grant FKZ 031A533 within the de.NBI network. Open access funding provided by University Library, Zurich University of Applied Sciences.

## Conflict of Interest

The authors declare that the research was conducted in the absence of any commercial or financial relationships that could be construed as a potential conflict of interest.

## Publisher’s Note

All claims expressed in this article are solely those of the authors and do not necessarily represent those of their affiliated organizations, or those of the publisher, the editors and the reviewers. Any product that may be evaluated in this article, or claim that may be made by its manufacturer, is not guaranteed or endorsed by the publisher.

## Supplementary Material

The Supplementary Material for this article can be found online at: https://www.frontiersin.org/articles/10.3389/fmicb.2022.804681/full#supplementary-material
