# Supplementary material for: Comparative Genomics of Prunus-Associated Members of the Pseudomonas syringae Species Complex Reveals Traits Supporting Co-evolution and Host Adaptation
Source: Front Microbiol. 2022 May 3;13:804681. doi: 10.3389/fmicb.2022.804681 (PMC9111521; doi:10.3389/fmicb.2022.804681)
Supplement: Supplementary file 1 [file Data_Sheet_1.PDF]

Supplementary Material

belonging to

Comparative genomics of *Prunus* associated members of the *Pseudomonas syringae* species complex reveals traits supporting co-evolution and host adaptation

Michela Ruinelli, Jochen Blom, Theo H.M. Smits and Joël F. Pothier

1 Supplementary Figures:

Supplementary Figure 1 | Average nucleotide identity (ANI) matrix and derived phylogeny of a set of 13 *Pseudomonas syringae* genomes.

Supplementary Figure 2 | Likelihood ratio (LR) distribution for the genes found to be shared at least between two genome subsets.

Supplementary Figure 3 | Comparison of the genomic context of nine of the 13 genes considered to be significantly associated with *Prunus* spp. based on 11 *Pseudomonas syringae* complete genomes.

Supplementary Figure 4 | Distribution profile of 13 proteins considered to be significantly associated with *Prunus* spp. among 13 additional *Pseudomonas* species closely related to the *Pseudomonas syringae* species complex.

Supplementary Figure 5 | HopAY and HopAR distribution based on *hopAY* and *hopAR* retrieval with BLASTN.

Supplementary Figure 6 | Alignment of the C58 cysteine proteases HopAR and HopAY.

2 Supplementary Tables:

Supplementary Table 1 | List of PBS1 protein sequences compared in this study.

3 Supplementary References

1 Supplementary Figures

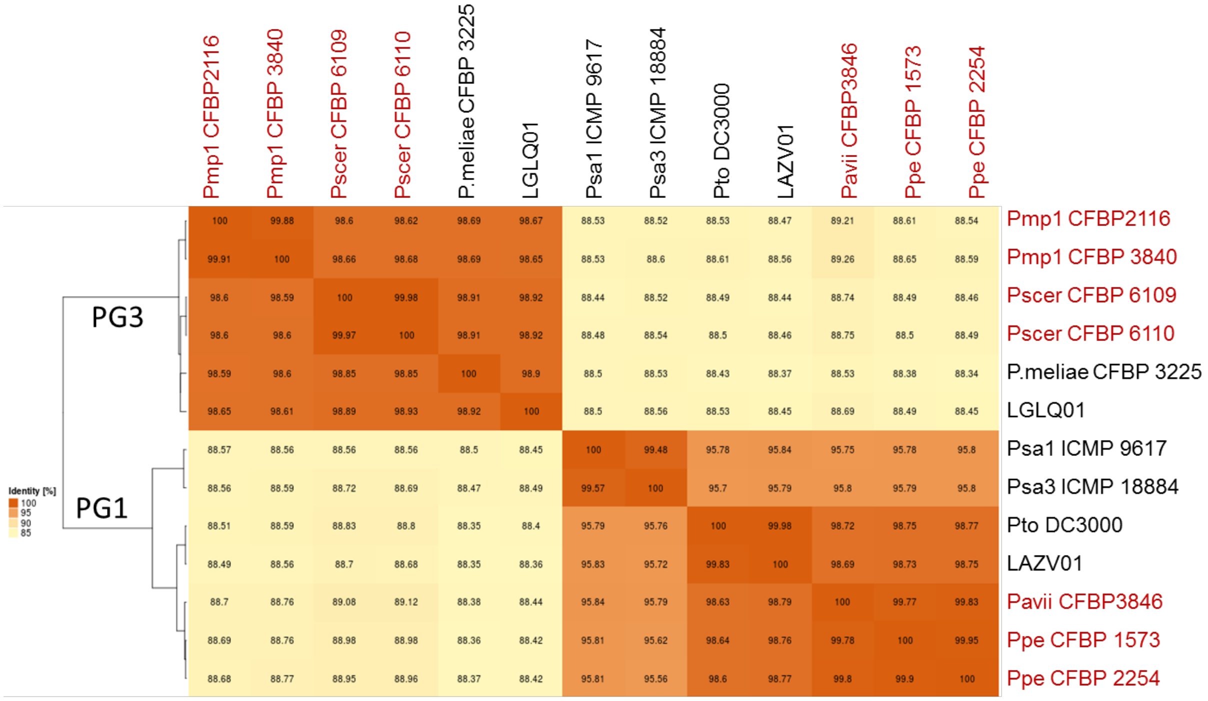

Supplementary Figure 1. Average nucleotide identity (ANI) matrix and derived phylogeny of a set of 13 *Pseudomonas syringae* genomes. Strains were selected based on their core-genome phylogeny relationships to the NCBI GenBank WGS accession prefixes LAZV01 (*P. syringae* pv. *persicae* NCPPB 2254) and LGLQ01 (*P. syringae* pv. *morsprunorum* race 1 HRI W 5269), genomes suspected to be misnamed. Strains isolated from *Prunus* sp. and sequenced by Ruinelli *et al.* (Ruinelli et al., 2019) are written in red. Numbers indicate the ANI percent identities among the pair of strains reported on the top and on the right. ANI threshold for species definition is 95%. Within the *P. syringae* species complex this value corresponds to the phylogroup (PG) boundaries. . Pmp1: *P. syringae* pv. morsprunorum race 1; Pscer: *P. syringae* pv. cerasicola; Psa1: *P. syringae* pv. actinidiae biovar 1; Psa3: *P. syringae* pv. actinidiae biovar 3; Pto: *P. syringae* pv. tomato; Pavii: *P. syringae* pv. avii; Ppe: *P. syringae* pv. persicae. ANI matrix and figure were generated using EDGAR v.2.2 (Blom et al., 2016).

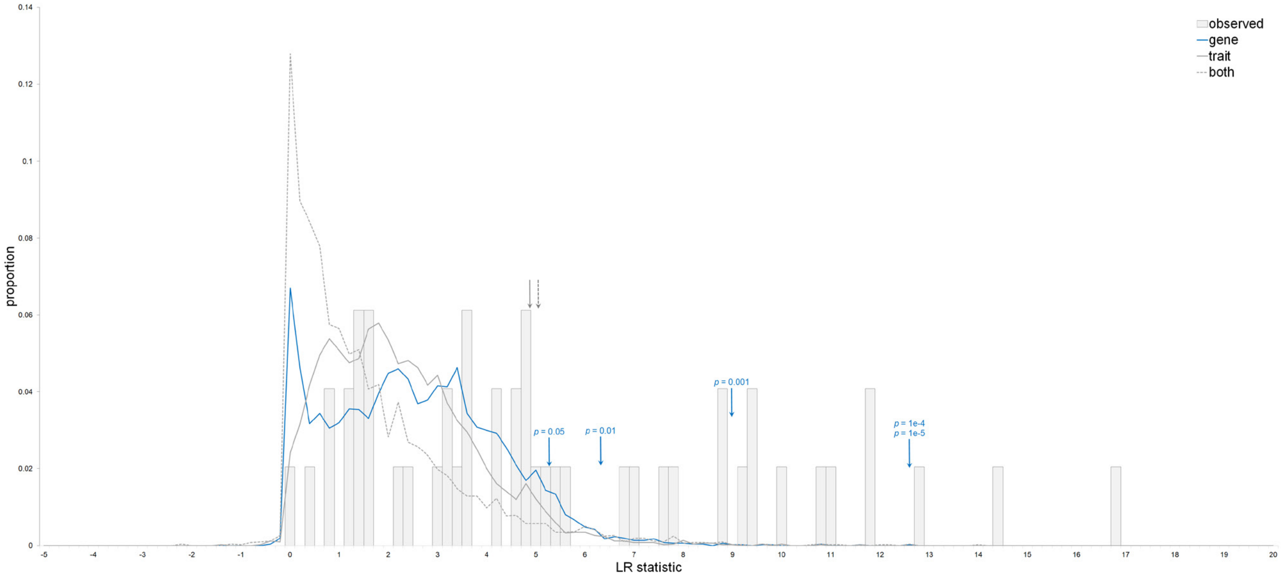

Supplementary Figure 2. Likelihood ratio (LR) distribution for the genes found to be shared at least between two genome subsets. Grey bars show the distribution of LR statistics for 49 tested sites found to be shared at least between two genome subsets designated A-D measuring the support for correlated evolution between *Prunus* spp. host isolation and gene presence. The estimated null based on permuting the gene occurrence data is overlaid in blue; arrows indicate the *p*-value thresholds based on this null. Also shown are two alternative null models, where either the trait data (solid light gray) or both trait data and gene occurrence (dashed light gray) have been randomized; the 5% *p*-value thresholds are also indicated with arrows for these alternative null distributions, for comparison.

A

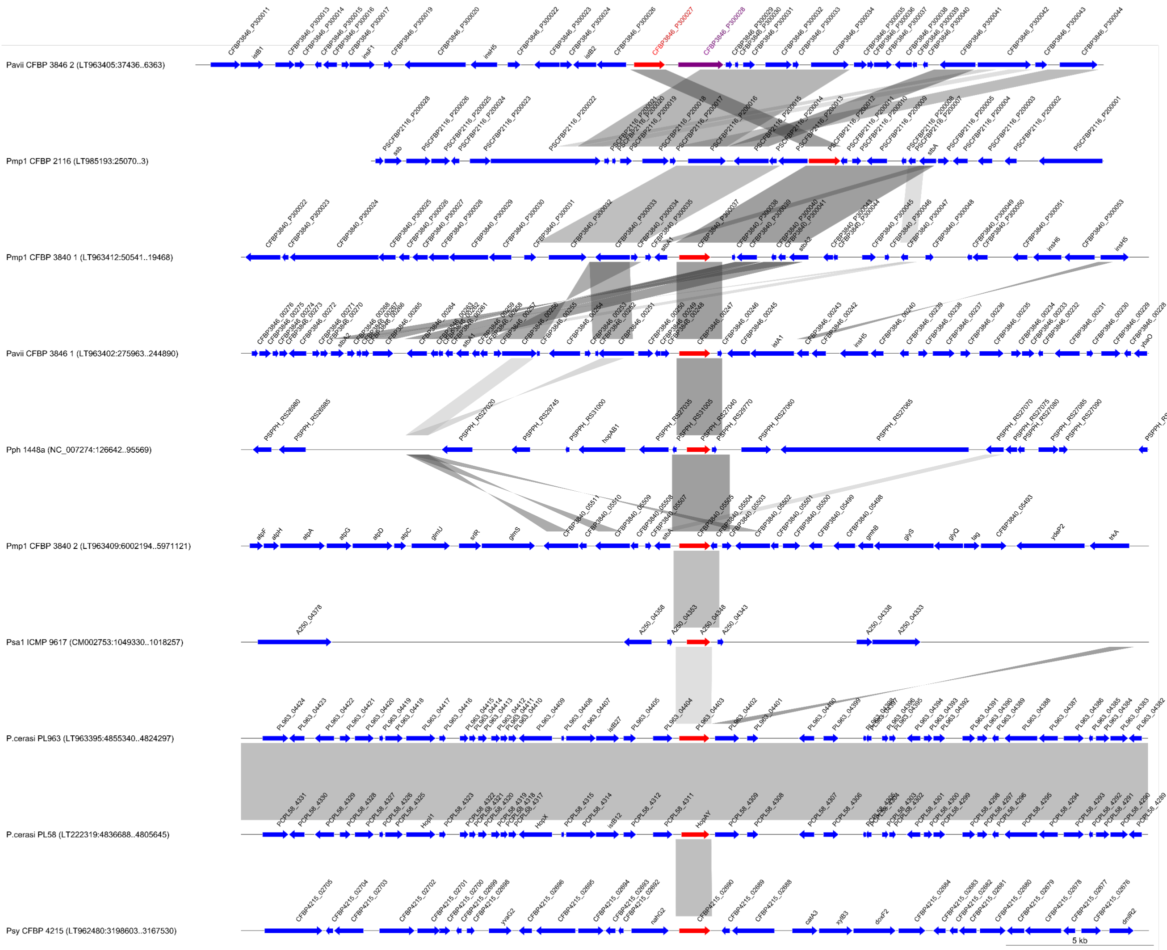
Pavii CFBP 3846 2 (LT963405:37436..6363)
Pmp1 CFBP 2116 (LT985193:25070..3)
Pmp1 CFBP 3840 1 (LT963412:50541..19468)
Pavii CFBP 3846 1 (LT963402:275963..244890)
Pph 1448a (NC_007274:126642..95569)
Pmp1 CFBP 3840 2 (LT963409:6002194..5971121)
Psa1 ICMP 9617 (CM002753:1049330..1018257)
P.cerasi PL963 (LT963395:4855340..4824297)
P.cerasi PL58 (LT222319:4836688..4805645)
Psy CFBP 4215 (LT962480:3198603..3167530)
5 kb

B

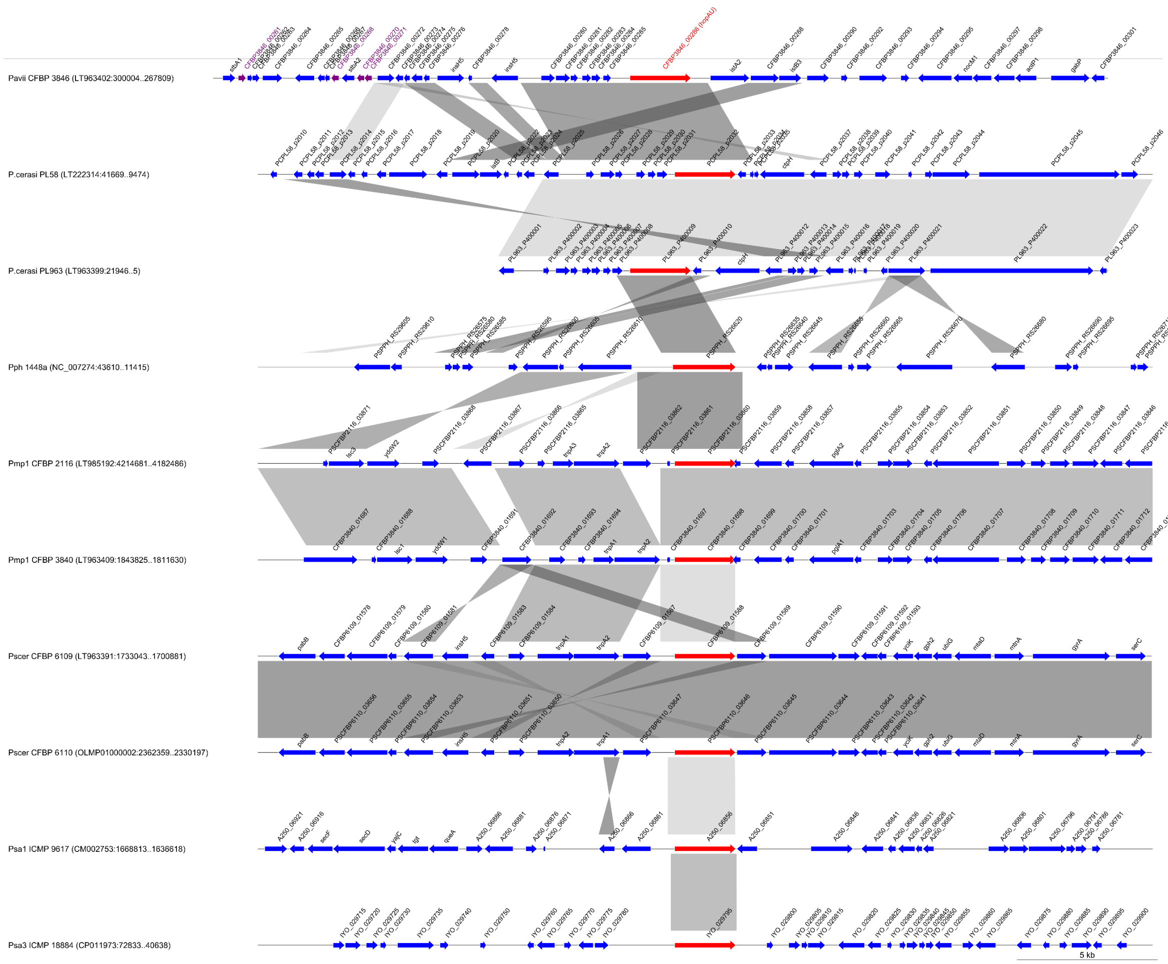
Pavii CFBP 3846 (LT963402:300004..267809)
P.cerasi PL58 (LT222314:41669..9474)
P.cerasi PL963 (LT963399:21946..5)
Pph 1448a (NC_007274:43610..11415)
Pmp1 CFBP 2116 (LT985192:4214681..4182486)
Pmp1 CFBP 3840 (LT963409:1843825..1811630)
Pscer CFBP 6109 (LT963391:1733043..1700881)
Pscer CFBP 6110 (OLMP01000002:2362359..2330197)
Psa1 ICMP 9617 (CM002753:1668813..1636618)
Psa3 ICMP 18884 (CP011973:72833..40638)
5 kb

C

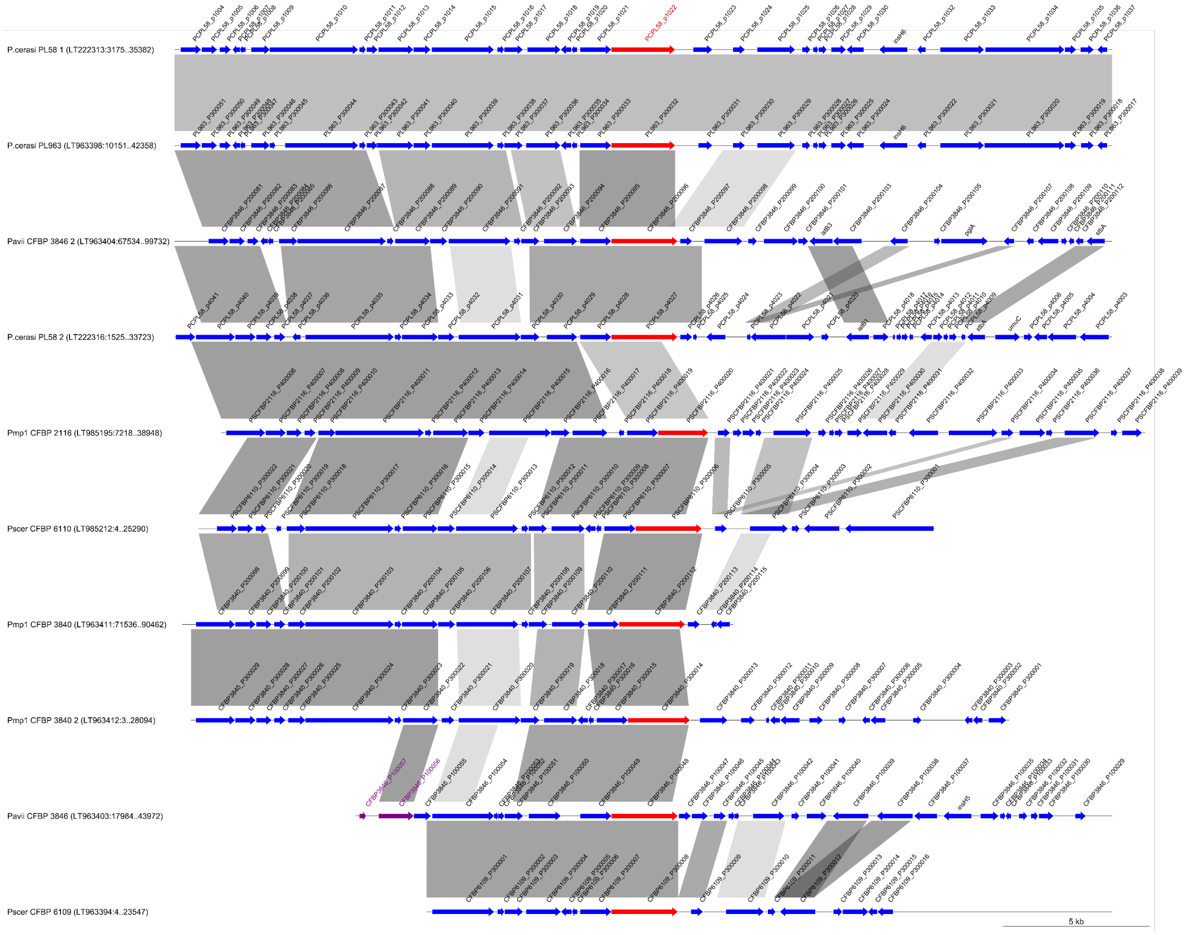
P.cerasi PL58 1 (LT222313:3175..35382)
P.cerasi PL963 (LT963398:10151..42358)
Pavii CFBP 3846 2 (LT963404:67534..99732)
P.cerasi PL58 2 (LT222316:1525..33723)
Pmp1 CFBP 2116 (LT985195:7218..38948)
Pscer CFBP 6110 (LT985212:4..25290)
Pmp1 CFBP 3840 (LT963411:71536..90462)
Pmp1 CFBP 3840 2 (LT963412:3..28094)
Pavii CFBP 3846 (LT963403:17984..43972)
Pscer CFBP 6109 (LT963394:4..23547)
5 kb

D

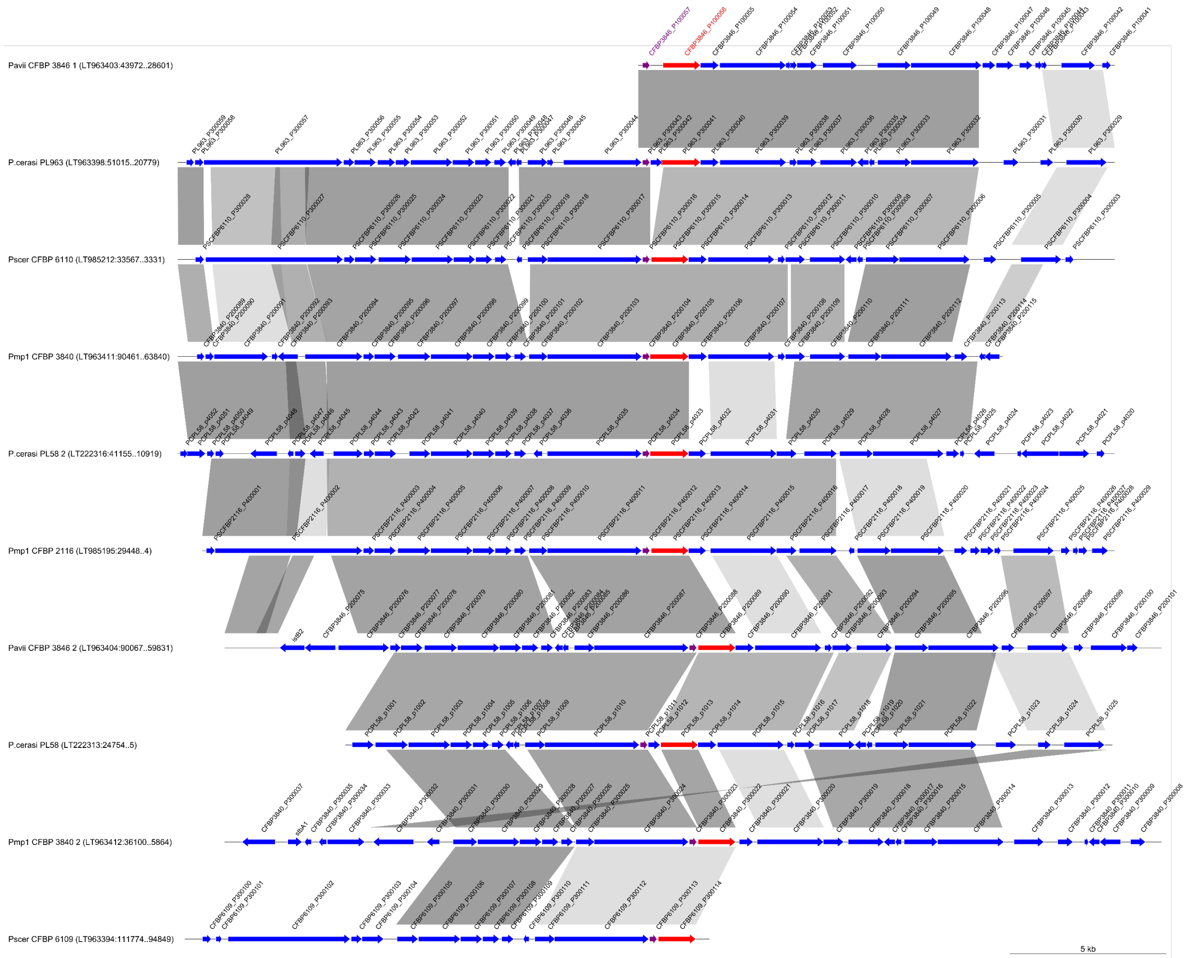

Pavii CFBP 3846 1 (LT963403:43972..28601)
P.cerasi PL963 (LT963398:51015..20779)
Pscer CFBP 6110 (LT985212:33567..3331)
Pmp1 CFBP 3840 (LT963411:90461..63840)
P.cerasi PL58 2 (LT222316:41155..10919)
Pmp1 CFBP 2116 (LT985195:29448..4)
Pavii CFBP 3846 2 (LT963404:90067..59831)
P.cerasi PL58 (LT222313:24754..5)
Pmp1 CFBP 3840 2 (LT963412:36100..5864)
Pscer CFBP 6109 (LT963394:111774..94849)
5 kb

E

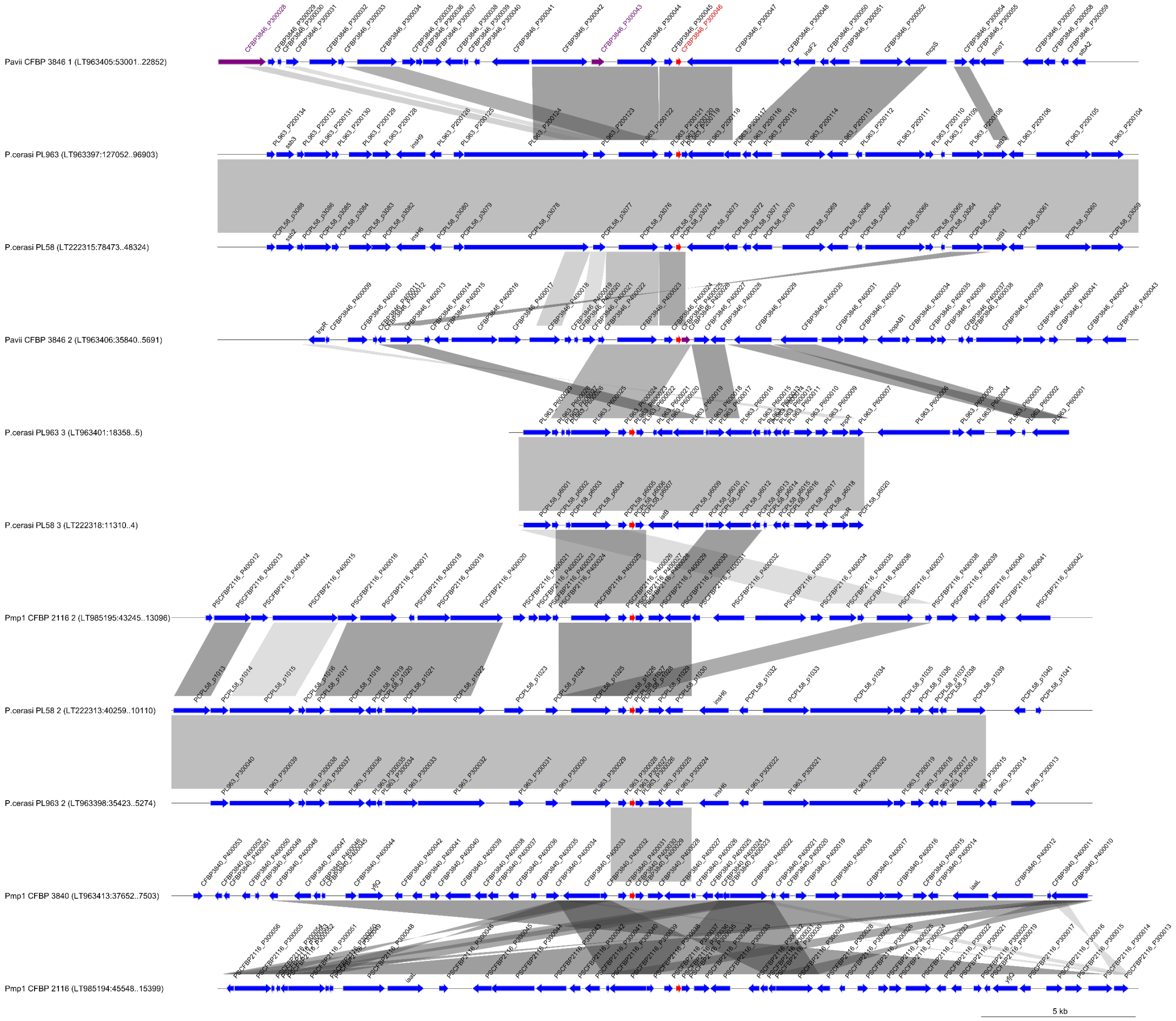
Pavii CFBP 3846 1 (LT963405:53001..22852)
P.cerasi PL963 (LT963397:127052..96903)
P.cerasi PL58 (LT222315:78473..48324)
Pavii CFBP 3846 2 (LT963406:35840..5691)
P.cerasi PL963 3 (LT963401:18358..5)
P.cerasi PL58 3 (LT222318:11310..4)
Pmp1 CFBP 2116 2 (LT985195:43245..13096)
P.cerasi PL58 2 (LT222313:40259..10110)
P.cerasi PL963 2 (LT963398:35423..5274)
Pmp1 CFBP 3840 (LT963413:37652..7503)
Pmp1 CFBP 2116 (LT985194:45548..15399)
5 kb

F

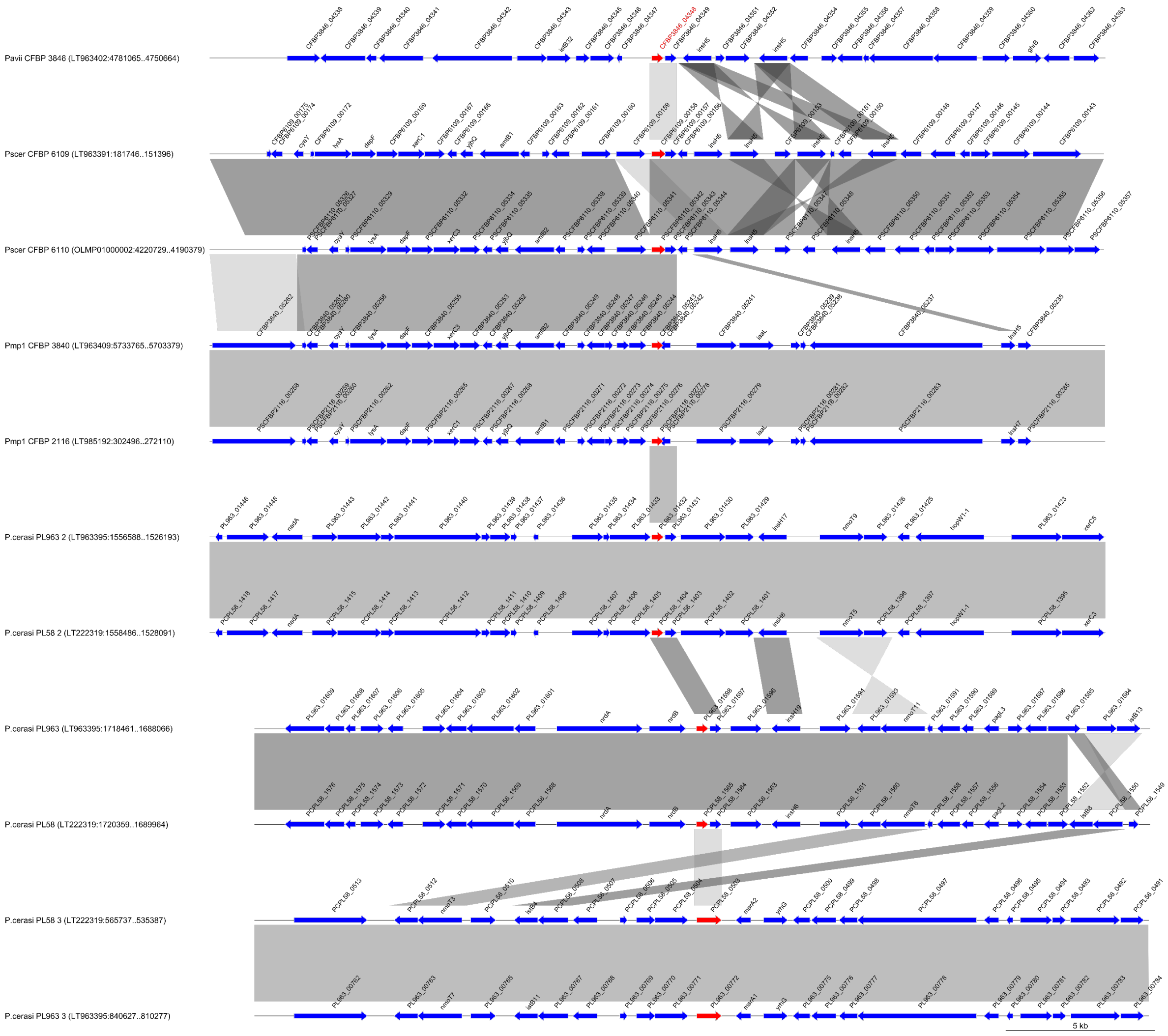

Pavii CFBP 3846 (LT963402:4781065..4750664)
Pscer CFBP 6109 (LT963391:181746..151396)
Pscer CFBP 6110 (OLMP01000002:4220729..4190379)
Pmp1 CFBP 3840 (LT963409:5733765..5703379)
Pmp1 CFBP 2116 (LT985192:302496..272110)
P.cerasi PL963 2 (LT963395:1556588..1526193)
P.cerasi PL58 2 (LT222319:1558486..1528091)
P.cerasi PL963 (LT963395:1718461..1688066)
P.cerasi PL58 (LT222319:1720359..1689964)
P.cerasi PL58 3 (LT222319:565737..535387)
P.cerasi PL963 3 (LT963395:840627..810277)
5 kb

G

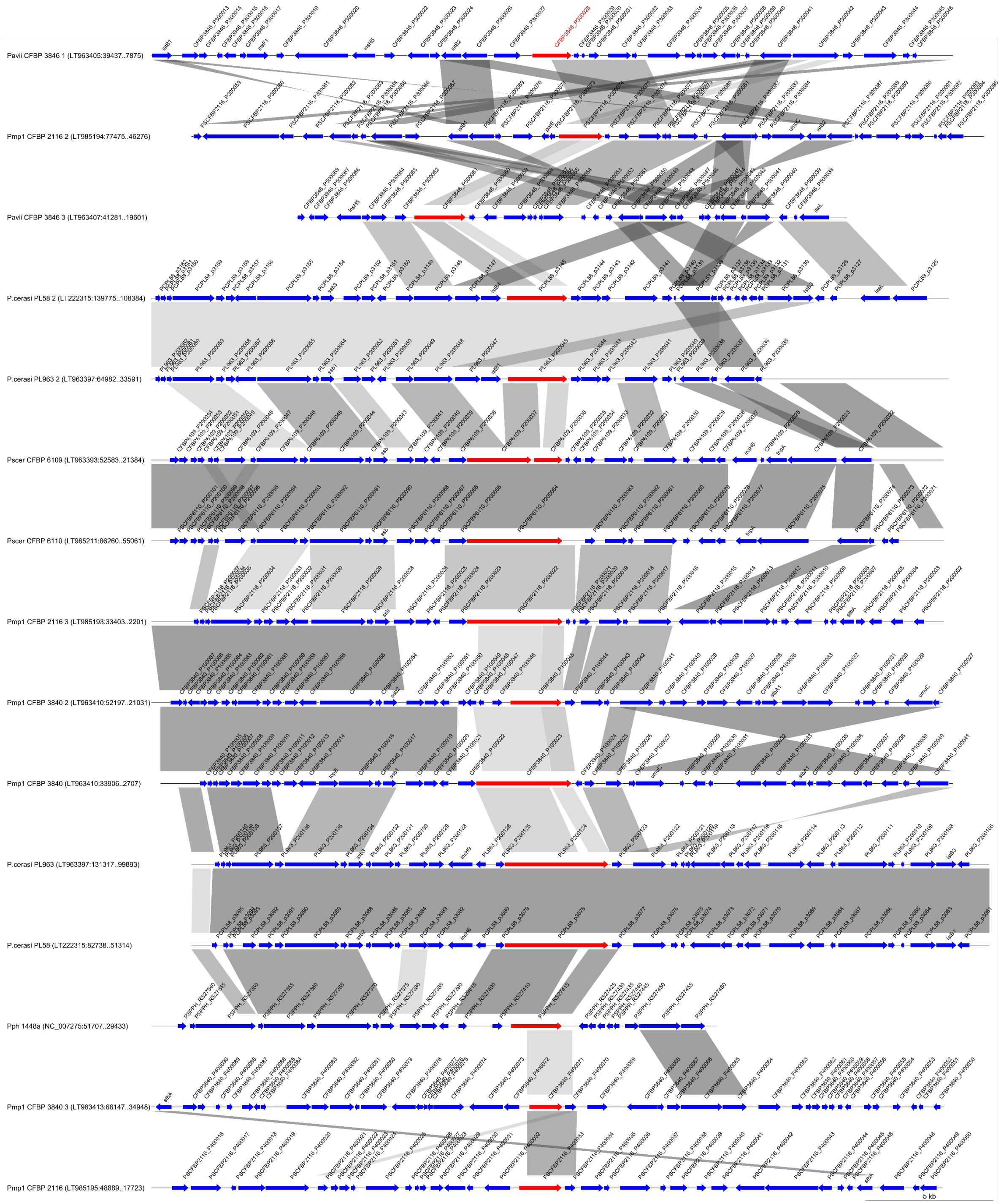
Pavii CFBP 3846 1 (LT963405:39437..7875)
Pmp1 CFBP 2116 2 (LT985194:77475..46276)
Pavii CFBP 3846 3 (LT963407:41281..19601)
P.cerasi PL58 2 (LT222315:139775..108384)
P.cerasi PL963 2 (LT963397:64982..33591)
Pscer CFBP 6109 (LT963393:52583..21384)
Pscer CFBP 6110 (LT985211:86260..55061)
Pmp1 CFBP 2116 3 (LT985193:33403..2201)
Pmp1 CFBP 3840 2 (LT963410:52197..21031)
Pmp1 CFBP 3840 (LT963410:33906..2707)
P.cerasi PL963 (LT963397:131317..99893)
P.cerasi PL58 (LT222315:82738..51314)
Pph 1448a (NC_007275:51707..29433)
Pmp1 CFBP 3840 3 (LT963413:66147..34948)
Pmp1 CFBP 2116 (LT985195:48889..17723)
5 kb

H

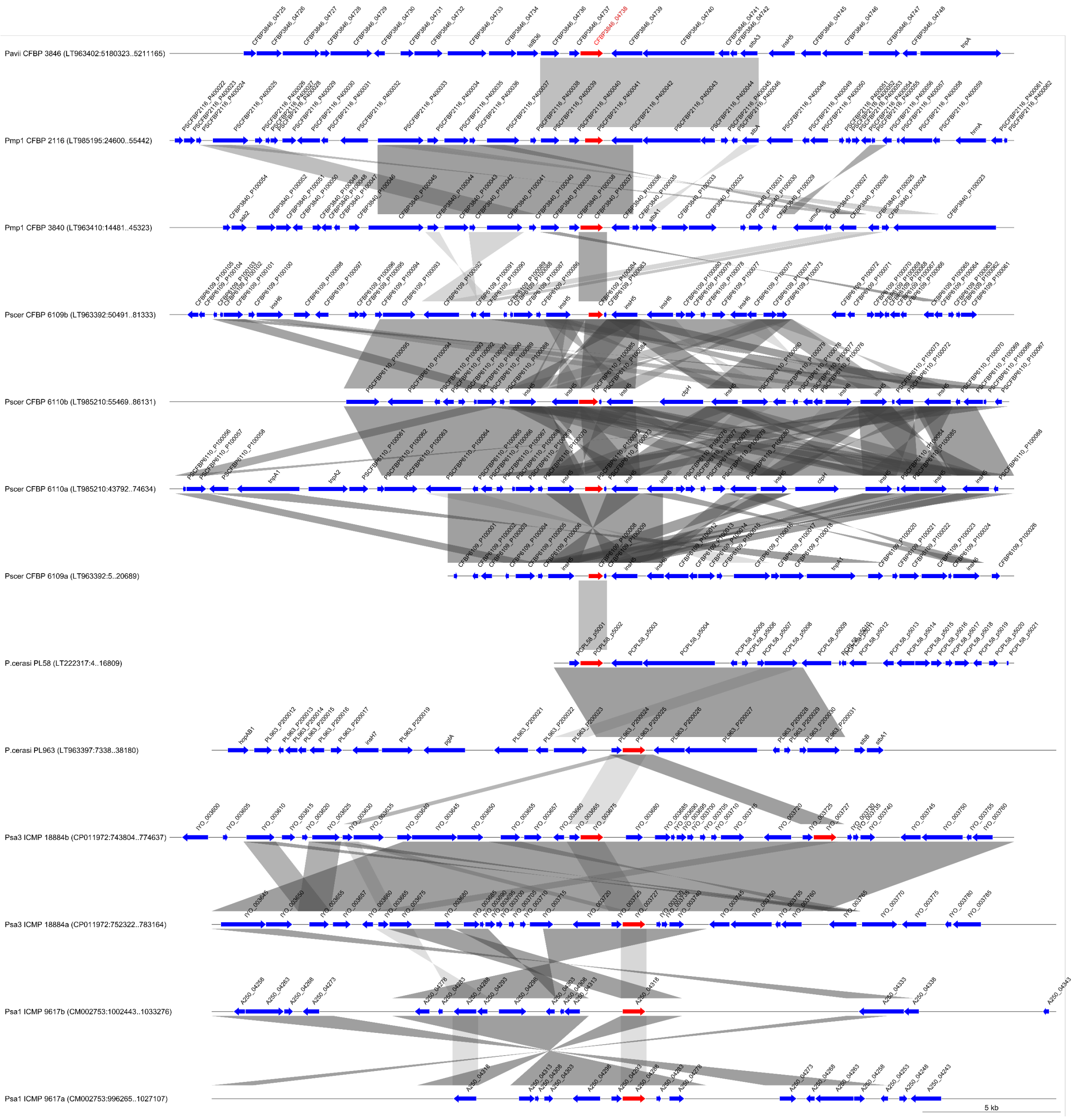
Pavii CFBP 3846 (LT963402:5180323..5211165)
Pmp1 CFBP 2116 (LT985195:24600..55442)
Pmp1 CFBP 3840 (LT963410:14481..45323)
Pscer CFBP 6109b (LT963392:50491..81333)
Pscer CFBP 6110b (LT985210:55469..86131)
Pscer CFBP 6110a (LT985210:43792..74634)
Pscer CFBP 6109a (LT963392:5..20689)
P.cerasi PL58 (LT222317:4..16809)
P.cerasi PL963 (LT963397:7338..38180)
Psa3 ICMP 18884b (CP011972:743804..774637)
Psa3 ICMP 18884a (CP011972:752322..783164)
Psa1 ICMP 9617b (CM002753:1002443..1033276)
Psa1 ICMP 9617a (CM002753:996265..1027107)
5 kb

I

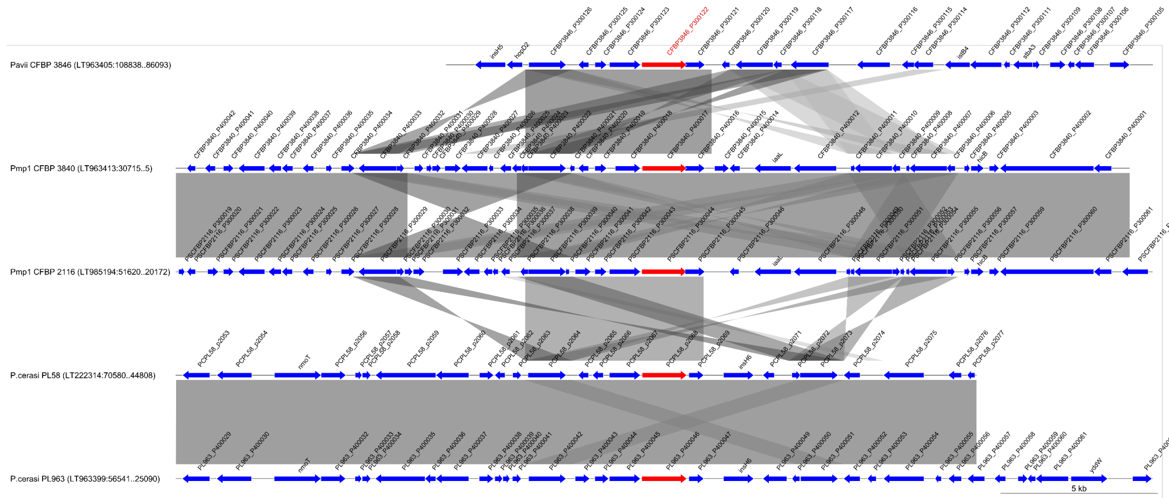

Supplementary Figure 3. Comparison of the genomic context of nine of the 13 genes considered to be significantly associated with *Prunus* spp. based on 11 *Pseudomonas syringae* complete genomes. The gene corresponding to the query used in the TBLASTN analysis (**Figure 1** and **Supplementary Figure 4**) is represented by a red arrow on the genome context located on top as well as in the other genome contexts. When present on the same genome context, other genes considered to be significantly associated with *Prunus* spp. are displayed with a purple arrow. Other genes are plotted with blue arrows which do not consider any shared identity between them. Regions with high DNA sequence identity are represented with grey blocks. The strain names used refer to the code field from **Table 1**. The strain name is followed by the GenBank accession number used and the locations displayed on the right. Gene annotation is mentioned on the top of each sequence. **(A)** CFBP_P30027 (HopAY). **(B)** CFBP3846_00286 (HopAU). **(C)** PCPL58_p1022 (TrbC protein). **(D)** CFBP3846_P10056 (conjugal transfer protein). **(E)** CFBP3846_P300046 (hypothetical protein, stability determinant). **(F)** CFBP3846_04348 (transposase). **(G)** CFBP3846_P30028 (relaxase). **(H)** CFBP3846_04738 (HopBB1). **(I)** CFBP3846_P300122 (FAD dependent oxidoreductase).

Organism (NCBI taxid)	NCBI BLAST database (amount of WGS)[a]	HopAY	h.p. (plasmid stabilization	transposase	h.p. (Ribbon-helix-helix protein)	h.p. (stability determinant)	FAD dependent oxidoreductase	h.p.	HopAU	trbC protein	HopBB1	conjugal transfer protein	glutathione S transferase	relaxase	Total number of hits	Proportion (%)
	LR	16.9738	14.51417	12.91178	11.8532	11.81981	10.92049	9.432982	8.888358	8.811136	7.908052	7.7251	7.082418	5.407748		
		CFBP3846_P300027	*CFBP3846_00270*	*CFBP3846_04348*	*CFBP3846_00271*	*CFBP3846_P300046*	*CFBP3846_P300122*	*CFBP3846_P100057*	*CFBP3846_00286*	*PCPL58_p1022*	*CFBP3846_04738*	*CFBP3846_P100056*	*PCPL58_4325*	*CFBP3846_P300028*		
Pseudomonas fluorescens (taxid:294)	WGS (250)		■	■	■										3	23.08
	refseq_genomes		■	■	■								■		4	30.77
Pseudomonas gessardii (taxid:78544)	WGS (8)														0	0.00
	refseq_genomes														0	0.00
Pseudomonas fragi (taxid:296)	WGS (31)		■		■										2	15.38
	refseq_genomes		■		■										2	15.38
Pseudomonas mandelii (taxid:75612)	WGS (5)														0	0.00
	refseq_genomes														0	0.00
Pseudomonas koreensis (taxid:198620)	WGS (20)		■		■								■		3	23.08
	refseq_genomes		■		■								■		3	23.08
Pseudomonas jessenii (taxid:77298)	WGS (6)		■		■								■		3	23.08
	refseq_genomes		■		■								■		3	23.08
Pseudomonas corrugata (taxid:47879)	WGS (10)														0	0.00
	refseq_genomes														0	0.00
Pseudomonas chlororaphis (taxid:587753)	WGS (31)												■		1	7.69
	refseq_genomes		■		■								■		3	23.08
Pseudomonas asplenii (taxid:53407)	WGS (5)		■		■								■		3	23.08
	refseq_genomes		■		■								■		3	23.08
Pseudomonas lutea (taxid:243924)	WGS (4)			■											1	7.69
	refseq_genomes			■											1	7.69
Pseudomonas syringae (taxid:317)	WGS (469)	■	■	■	■	■	■	■	■	■	■	■	■	■	13	100.00
	refseq_genomes	■	■	■	■	■	■	■	■	■	■	■	■	■	13	100.00
Pseudomonas putida (taxid:303)	WGS (139)		■		■								■		3	23.08
	refseq_genomes		■		■								■		3	23.08
Pseudomonas straminea (taxid:47882)	WGS (1)														0	0.00
	refseq_genomes														0	0.00
Pseudomonas anguilliseptica (taxid:53406)	WGS (1)														0	0.00
	refseq_genomes														0	0.00

[a] Database version 2021/01/26.

Supplementary Figure 4. Distribution profile of 13 proteins considered to be significantly associated with *Prunus* spp. among 13 additional *Pseudomonas* species closely related to the *Pseudomonas syringae* species complex. Black indicates presence of at least one tBLASTn hit with 70% identity and 70% subject coverage whereas white indicates absence of a hit. The 13 proteins are ordered by decreasing significance of the likelihood ratio (LR) statistic. Locus tags are reported over each considered protein; h.p.: hypothetical protein. Proteins highlighted in pink are involved in virulence based on their annotation.

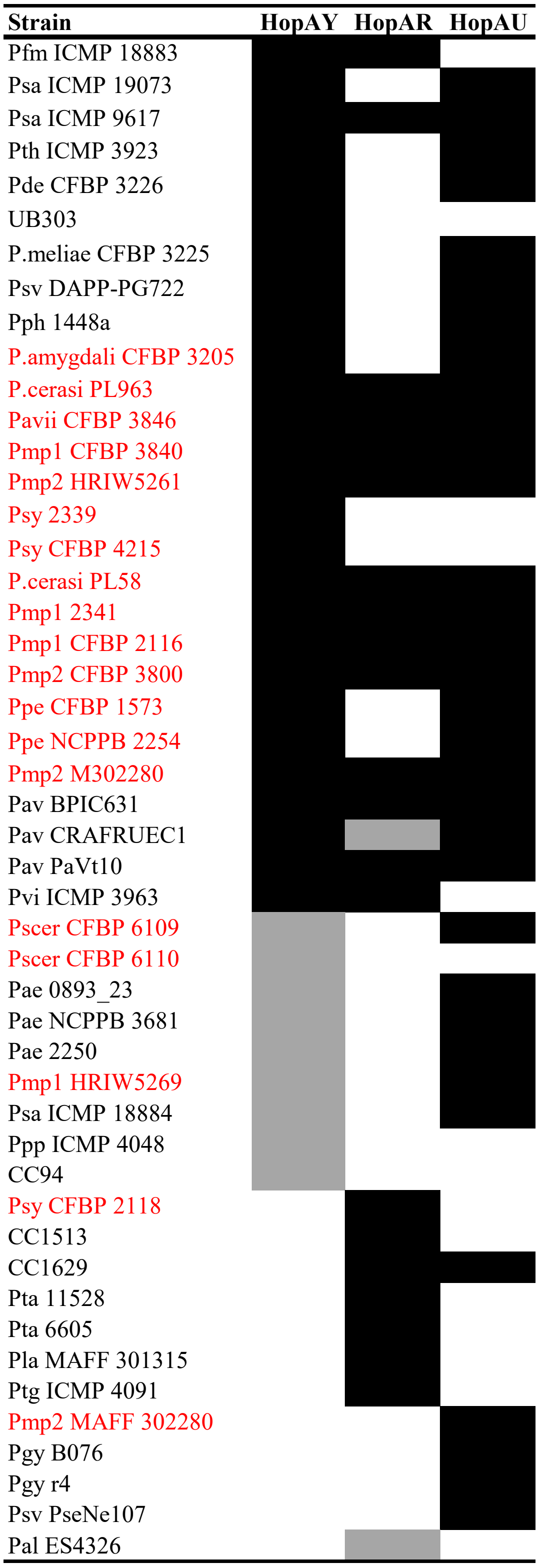

Supplementary Figure 5. HopAY, HopAR and HopAU distribution based on *hopAY*, *hopAR* and *hopAU* retrieval with BLASTN. Strains in red were isolated from *Prunus* spp. Abbreviations corresponds to **Table 1** in the main document. Black indicates presence of a full-length gene whereas grey indicates presence of a truncated gene. White indicates absence of the gene.

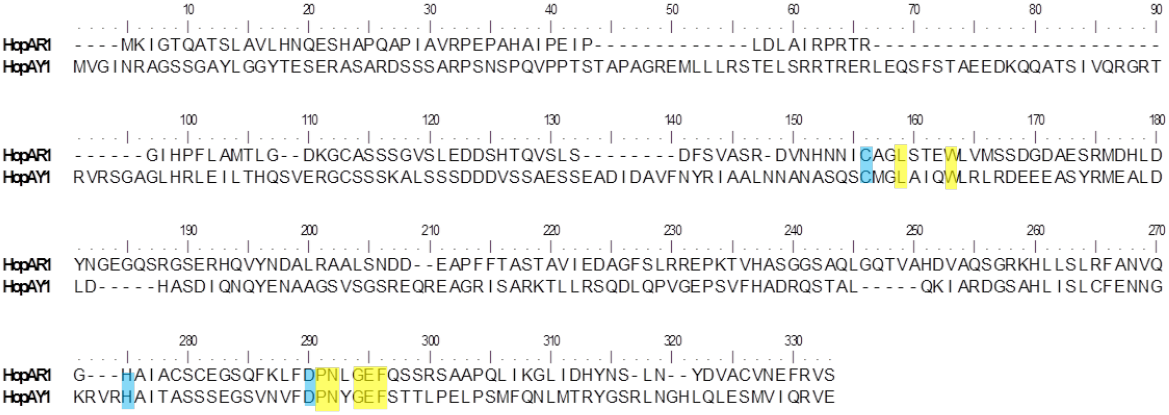

Supplementary Figure 6. Alignment of the C58 cysteine proteases HopAR and HopAY. Blue residues indicate the catalytic triad consisting of a cysteine (C), a histidine (H) and an aspartate (D) which are typical for proteins of the C58 peptidase family. Yellow blocks represent residues shown to be conserved among members of the C58 peptidase family within different bacteria (Zhu et al., 2004). Both HopAR and HopAY sequences were retrieved from the *Pseudomonas syringae* Hop database (www.pseudomonas-syringae.org) and are belonging to the *P. syringae* pv. phaseolicola race 3 (GenBank accession number AAA25727) and strain 1448a (GenBank locus tag PSPPH_A0129), respectively. Alignment was performed using ClustalW within MEGA 6.0.

2 Supplementary Tables

Supplementary Table 1. List of PBS1 protein sequences compared in this study.

Plant species	Plant family	GenBank Acc. Nr.	NCBI database
Actinidia chinensis [1]	*Actinidiaceae*	GAMA01014302	TSA
Dendropanax trifidus [1]	*Araliaceae*	N.A.	N.A.
Panax ginseng [3]	*Araliaceae*	GDQW01078935	TSA
Corylus avellana [1]	*Betulaceae*	KA397503.1	TSA
Arabidopsis thaliana	*Brassicaceae*	NP_196820	Protein
Capsella rubella	*Brassicaceae*	EOA20310	Protein
Cucumis melo [1]	*Cucurbitaceae*	XM_008461668.2	TSA
Cucumis sativusa	*Cucurbitaceae*	XP_004140546	Protein
Ricinus communis	*Euphorbiaceae*	XP_002514864	Protein
Cicer arietinum	*Fabaceae*	XP_004497430	Protein
Glycine max	*Fabaceae*	NP_001235164	Protein
Glycine max cv. Williams 82	*Fabaceae*	XP_003556585	Protein
Phaseolus vulgaris [1]	*Fabaceae*	XP_007142596.1	Protein
Olea europea [1]	*Oleaceae*	GBKW01050313.1	TSA
Setaria italica	*Poaceae*	XP_004952572	Protein
Sorghum bicolor	*Poaceae*	XP_002452162	Protein
Zea mays	*Poaceae*	NP_001149465	Protein

Crataegus pinnatifida	*Rosaceae*	GALU01004321.1	TSA
Fragaria vesca subsp. vesca	*Rosaceae*	XP_004310112	Protein
Malus × domestica [1]	*Rosaceae*	XM_008391700.2	TSA
Prunus armeniaca	*Rosaceae*	GAWA01009274.1	TSA
Prunus avium [1]	*Rosaceae*	GAJZ01006901.1	TSA
Prunus mume	*Rosaceae*	XP_008222220.1	Protein
Prunus persica [1]	*Rosaceae*	XP_007225732.1	Protein
Pyrus pyrifolia	*Rosaceae*	GALR01009739.1	TSA
Rubus sp.	*Rosaceae*	HACC01011112.1	TSA
Populus trichocarpa	*Salicaceae*	XP_002304664	Protein
Aesculus hyppocastanum [1]	*Sapindaceae*	N.A.	N.A.
Litchi chinensi [2]	*Sapindaceae*	GCAD01038413.1	TSA
Solanum lycopersicum	*Solanaceae*	XP_004239305	Protein
Theobroma cacao	*Sterculiaceae*	EOX99706	Protein
Camellia sinensis [1]	*Theaceae*	GEFQ01040965.1	TSA
Vitis vinifera	*Vitaceae*	XP_002265076	Protein

[1] At least one strain associated with this plant species possessed *hopAY*.
[2] Same family as *Aesculus hyppocastanum* from which *Pseudomonas amygdali* pv. *aesculi* strains possessing a truncated HopAY were isolated.
[3] Same family as *Dendropanax trifidus* from which the HopAY possessing strain *P. amygdali* pv. dendropanacis CFBP 3226 was isolated.

TSA: Transcriptome Shotgun Assembly Sequence; N.A.: not available.

3 Supplementary References

Blom, J., Kreis, J., Spänig, S., Juhre, T., Bertelli, C., Ernst, C., et al. (2016). EDGAR 2.0: an enhanced software platform for comparative gene content analyses. *Nucleic Acids Research* 44**,** W22-28. doi: 10.1093/nar/gkw255.

Ruinelli, M., Blom, J., Smits, T.H.M., and Pothier, J.F. (2019). Comparative genomics and pathogenicity potential of members of the *Pseudomonas syringae* species complex on *Prunus* spp. *BMC Genomics* 20(1)**,** 172. doi: 10.1186/s12864-019-5555-y.

Zhu, M., Shao, F., Innes, R.W., Dixon, J.E., and Xu, Z. (2004). The crystal structure of *Pseudomonas* avirulence protein AvrPphB: A papain-like fold with a distinct substrate-binding site. *Proceedings of the National Academy of Sciences, USA* 101(1)**,** 302-307. doi: 10.1073/pnas.2036536100.
